# TET2 orchestrates YAP signaling to potentiate targetable vulnerability in hepatocellular carcinoma

**DOI:** 10.1038/s41419-025-07745-3

**Published:** 2025-06-05

**Authors:** Jing He, Mingen Lin, Fei Teng, Xue Sun, Ziyin Tian, Jiaxi Li, Yan Ma, Yue Dai, Yi Gao, Hongchen Li, Tongguan Tian, Kai Xu, Xinxing Li, Lei Lv, Yanping Xu

**Affiliations:** 1 Tongji Hospital, Shanghai Key Laboratory of Signaling and Disease Research, Frontier Science Center for Stem Cell Research, School of Life Sciences and Technology, Tongji University, Shanghai, China; 2 MOE Key Laboratory of Metabolism and Molecular Medicine, Department of Biochemistry and Molecular Biology, School of Basic Medical Sciences, Fudan University, Shanghai, China; 3 Department of Liver Surgery and Organ Transplantation, Changzheng Hospital, Naval Medical University, Shanghai, China; 4 Department of Gastrointestinal Surgery, Tongji Hospital Medical College of Tongji University, Shanghai, China

**Keywords:** Cancer therapeutic resistance, Growth factor signalling

## Abstract

Hepatocellular carcinoma (HCC) is a leading cause of global cancer-associated mortality. Although various therapies have substantially ameliorated clinical outcome, patients invariably suffer from cancer relapse, highlighting the need for more optimized therapeutic strategies. Here, we report that deficiency of DNA methylcytosine dioxygenase TET2 sensitizes HCC cells to sorafenib and verteporfin treatments. Mechanistically, knockout of TET2 enhances the dephosphorylation of YAP Ser127, thus promoting its activity. RNA-seq analysis reveals that MC1R, a GPCR, is strikingly decreased upon TET2 deficiency. Furthermore, TET2 catalyzes demethylation of MC1R promoter to stimulate its transcription. MC1R subsequently boosts cAMP-PKA signaling to phosphorylate YAP Ser127 in both ligand dependent and independent manners. Importantly, deletion of MC1R accelerates tumor growth of HCC, which is reversed by the treatment of YAP-TEAD complex inhibitor verteporfin. Synergistic combination of MC1R expression driver vitamin C and its ligand α-MSH dramatically represses HCC growth. Notably, TET2-MC1R-YAP axis is evidenced in HCC specimens and plays a vital role in prognosis of HCC. Collectively, these findings not only elucidate a previously unidentified epigenetic regulatory mechanism of MC1R transcription and underscore the functional significance of MC1R signaling in tumorigenesis of HCC, but also provide potential targets and clinical strategies for HCC therapy.

## Introduction

Liver cancer is the sixth frequently occurring cancer and third leading cause of cancer-associated mortality worldwide [1]. Hepatocellular carcinoma (HCC) represents about 75–85% of all primary liver cancer cases [1] and is mainly developed from virus infection, smoking, aflatoxin exposure, alcohol abuse and other metabolic syndromes [2]. Clinically, a plethora of surgical and pharmacological therapies are employed for the management of HCC. Early-stage HCC is amenable to potentially curative treatments such as resection, transplantation and ablation [3]. Nevertheless, patients bearing multiple symptoms are always diagnosed with advanced-stage HCC, which accounts for more than 50% of determined HCC [4, 5]. Sorafenib, a multi-kinase inhibitor approved by FDA, provides an alternative option for unresectable HCC [6]. Although patients receiving sorafenib substantially achieved extended survival by almost three months [6], one of the considerable hurdles for development of the systemic therapy is to identify the most responsive patient subsets and ameliorate drug resistance, since only approximately 35% patients benefit from sorafenib treatment with poor time to progression (TTP) [7]. Hence, those disadvantages of current clinical therapies highlight the need for more personalized approaches to improve the efficacy of HCC therapeutics.

Epigenetic dysfunction is a hallmark of cancer and contributes to adaptive resistance to anticancer drugs [8]. Combination therapies incorporating epigenetic inhibitors have been proved able to overcome acquired drug resistance of cancer cells [9]. For instance, HCC cells treated with sorafenib are characterized by divergent alterations in DNA methylation level of whole genome [10]. 5-azacytidine (5-AZA), the inhibitor of DNMTs, could improve the efficacy of sorafenib in HCC cells [11], which further underlines the crucial role of DNA methylation in sorafenib resistance of HCC. DNA demethylation is closely related with gene expression and mainly catalyzed by TET methylcytosine dioxygenases family through converting 5mC to 5hmC, then 5fC and 5caC [12]. Unlike TET1 and TET3, TET2 is frequently mutated in myeloid cancers [13], noting that most of these mutations impair its activity [14]. Deficiency of Tet2 originates aberrant self-renewal of hematopoietic stem cells [15] and subsequent onset of myeloid malignancies in mice [16], implying Tet2 functions as a tumor suppressor. Clinically, activation of TET2-mediated chemokine expression enhances immunotherapy [17], while loss of TET2 compromises the exceptional effect of vitamin C on VHL-deficient clear cell renal cell carcinoma [18]. Nonetheless, the functional role and mechanism of TET2 in drug resistance, especially sorafenib resistance of HCC, remain to be elucidated.

Hippo signaling pathway is an evolutionarily conserved serine/threonine kinase cascade, which is frequently altered in human cancers [19, 20]. YAP/TAZ are major effectors of Hippo pathway and function as master regulators of organ size and tissue homeostasis by coordinating cell proliferation and differentiation [21]. In response to a multitude of intrinsic and extrinsic signals, dephosphorylated YAP/TAZ translocate to nucleus and form a transcriptional complex with TEADs, thereby enabling gene expression [21, 22]. YAP exerts a dominant role in liver tumorigenesis since almost 60% of human liver cancer is accompanied with increased YAP activity [23]. Besides, Yap also acts as a potent driver gene of liver cancer in mouse model [24]. Lately, an increasing body of studies implicate that YAP/TAZ-mediated transcription could converge to adaptive resistance to chemotherapy, targeted therapy and immunotherapy [25, 26]. For instance, YAP stimulates AXL and EGFR transcription, and accordingly reinforces cellular resistance to EGFR inhibitor and docetaxcel in non-small cell lung cancer and esophageal cancer, respectively [27, 28]. Likewise, YAP promotes ACSL4 and TFRC transcription, which subsequently lead to ferroptosis [29]. It was reported that sorafenib functions as an inhibitor of cystine transporter SLC7A11 and also promotes ferroptosis [30], therefore, cancer cells with energetic YAP signaling are more sensitive to apoptosis induced by sorafenib [29]. Conversely, long-term sorafenib treatment could stimulate nuclear localization of YAP to upregulate the expression of SLC7A11 and Survivin, thus acquiring resistance to sorafenib [31, 32]. These evidences above highlight the critical spatiotemporal correlation between YAP and sorafenib. Considering that epigenetic drug booms as a rising star for cancer treatment [33], it is of importance to clarify the potential bridge role of YAP in crosstalk between epigenetics and sorafenib resistance of HCC.

In present study, we report that TET2 deficiency promotes YAP transcriptional activity and sensitizes HCC cells to sorafenib and verteporfin treatments. Through RNA-seq analysis screen, we found G-protein-coupled receptor MC1R mediates the regulation of YAP activity by TET2 via cAMP-PKA signaling. MC1R functions as a tumor suppressor by triggering phosphorylation of YAP and inhibiting its activity in both ligand dependent and independent manners. Eventually, we demonstrated vitamin C reinforces the anticancer effect of MC1R ligand α-MSH in vitro and in vivo and highlighted the prognostic significance of TET2-MC1R-YAP axis in HCC. Our findings not only put forth a heretofore unrecognized mechanism underlying the regulation of YAP signaling by TET2, but also identify TET2 as a potential biomarker for predicting sorafenib resistance of HCC, providing a possible route for precision therapy in clinic. Ultimately, we proposed a novel strategy by synergistic combination of α-MSH and vitamin C for HCC therapy.

## Results

### Deficiency of TET2 promotes YAP activity and sensitizes HCC cells to sorafenib and verteporfin

**Fig. 1:**
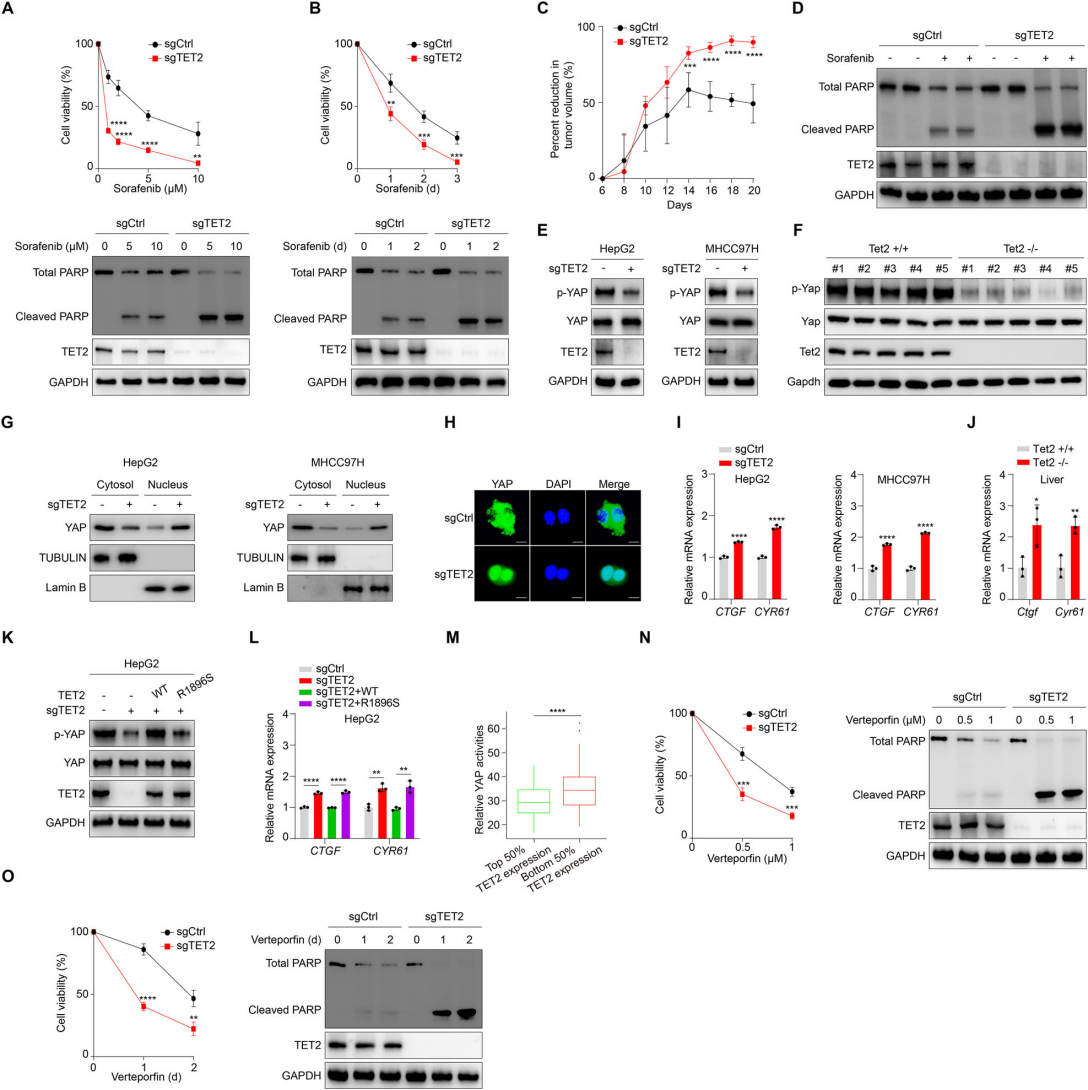
Deficiency of TET2 promotes YAP activity and sensitizes HCC cells to sorafenib and verteporfin. Deficiency of TET2 sensitizes HCC cells to sorafenib. Control and TET2 KO HepG2 cells were treated with different concentration of sorafenib as indicated for three days (**A**), or 10 μM sorafenib for different times as indicated (**B**). Cell viability was analyzed in top panels. Western blot analysis of cleaved PARP was performed in bottom panels. **C** TET2 deficiency sensitizes tumor cells to sorafenib treatment in vivo. Control and TET2 KO MHCC97H cells were subcutaneously injected into the left flanks of athymic nude mice (*n* = 6 per group). Tumor inhibition rate of sorafenib was measured and calculated every other day as indicated. **D** Western blot analysis of tumors from nude mice was performed with indicated antibodies. Phosphorylation level of YAP Ser127 is significantly downregulated in sgTET2 HCC cells (**E**) and livers of Tet2 knockout mice (**F**). **G** Western blot analysis of cytoplasm and nuclear extracts derived from Control and TET2 KO cell lines (HepG2 or MHCC97H). **H** Immunofluorescence of YAP localization in Control or TET2 KO HepG2 cells. YAP target genes *CTGF* and *CYR61* are distinctly upregulated in sgTET2 HCC cells (**I**) and livers of Tet2 knockout mice (**J**). **K** Re-introduction of WT TET2 not catalytic mutant TET2 (R1896S) can rescue phosphorylation level of YAP Ser127 in sgTET2 HepG2. **L** Reintroduction of WT TET2 can rescue mRNA expression of *CTGF* and *CYR61*. **M** A total of 253 LIHC tumors from TCGA database were divided into two groups based on TET2 mRNA levels (top and bottom 50% TET2 expression), and their relative YAP activities were qualified and plotted as described in the Methods. LIHC, liver hepatocellular carcinoma. **N**, **O** Deficiency of TET2 sensitizes HCC cells to verteporfin. Control and TET2 KO HepG2 cells were treated with different concentration of verteporfin as indicated for two days (**N**), or 1 μM verteporfin for different times as indicated (**O**). Cell viability was analyzed in top panels. Western blot analysis of cleaved PARP was performed in bottom panels. Data are presented as mean ± s.d., *n* = 3 independent repeats. Unpaired, two-tailed *t*-test.

In the absence of drug intervention, TET2 deficiency significantly enhances tumor cell viability in HCC cell lines (Supplementary Fig. S1A). To explore the potential significance of TET2 in drug resistance, cell viability was examined upon treatment with various drugs applied in HCC management clinically. Sorafenib, lenvatinib and regorafenib are broadly utilized in clincial practical treatment for HCC patients [3, 6, 34, 35]. Intriguingly, deficiency of TET2 sensitizes cell to sorafenib with dramatically decreased cell viability and elevated cleaved PARP, which is a biomarker of cell apoptosis [36] (Fig. 1A, B), whereas lenvatinib and regorafenib fail to exert similar effect in TET2 knockout (hereafter sgTET2) HCC cells (Supplementary Fig. S1B–E). To verify the effect in vivo, we subcutaneously injected control and sgTET2 HCC cells into athymic nude mice and employed sorafenib treatment after tumor initiation. Consistently, tumors with TET2 deficiency are prone to growth arrest upon sorafenib treatment (Fig. 1C). Immunoblotting analysis revealed that tumors lack of TET2 present higher level of cleaved PARP in response to sorafenib (Fig. 1D). Together, deficiency of TET2 sensitizes HCC to sorafenib treatment. Given the functional role of YAP in HCC tumorigenesis and drug resistance, we further tried to uncover the regulation of YAP by TET2 and found that the phosphorylation level of YAP Ser127 (hereafter p-YAP) is significantly downregulated in sgTET2 HCC cells (Fig. 1E) and liver tissues of Tet2 knockout mice (Fig. 1F). Concurrently, we also evaluated YAP subcellular localization and found that TET2 deficiency triggers translocation of YAP into the nucleus (Fig. 1G, H). By virtue of the phosphorylation of Ser127 being the common negative indicator for YAP activity, we next tested the expression of its target genes (*CTGF* and *CYR61*) in sgTET2 cells. As expected, *CTGF* and *CYR61* are distinctly upregulated in sgTET2 HCC cells (Fig. 1I) and livers of Tet2 knockout mice (Fig. 1J). Moreover, we also provided evidence that the regulation of YAP activity by TET2 is dependent on its catalytic activity via introducing wild-type TET2 (WT) and catalytic dead mutant (R1896S) to sgTET2 cells (Fig. 1K). Consistently, WT TET2, but not R1896S, rescues the expression of *CTGF* and *CYR61* in sgTET2 cells (Fig. 1L). Furthermore, we analyzed the correlation between TET2 expression and YAP activity in liver hepatocellular carcinoma (LIHC) derived from TCGA database. Interestingly, TET2 expression is negatively related with YAP activity in LIHC (Fig. 1M), implying the extensive existence of TET2-YAP axis in clinical samples. To validate the physiological roles of TET2 and YAP in HCC, we examined the sorafenib-resistant (RS) cells (Supplementary Fig. S1F) and found that the expression of YAP but not TET2 is substantially induced compared with wild-type cells (WT) (Supplementary Fig. S1F), which is consistent with previous findings [31]. Moreover, deletion of TET2 exerted minor effect on viability of sorafenib-resistant (RS) cells (Supplementary Fig. S1G), suggesting the context-dependent role of TET2 in modulating sorafenib sensitivity.

Cancer cells harbor constitutively activated oncogenic signals and may develop addiction to these signals to maintain sustainable proliferation [37, 38], we thus employed verteporfin, a YAP inhibitor, to investigate whether the viability of sgTET2 cells is even more dependent on YAP activation. Strikingly, sgTET2 cells present poor viability and higher level of cleaved PARP in response to verteporfin (Fig. 1N, O), rendering the evidence that sgTET2 cells may have evolved YAP addiction for proliferation, which can be apparently blocked by verteporfin treatment. Collectively, deficiency of TET2 promotes YAP activity and sensitizes HCC cells to sorafenib and verteporfin treatment.

### TET2 suppresses YAP activity via MC1R-cAMP-PKA signaling pathway

**Fig. 2:**
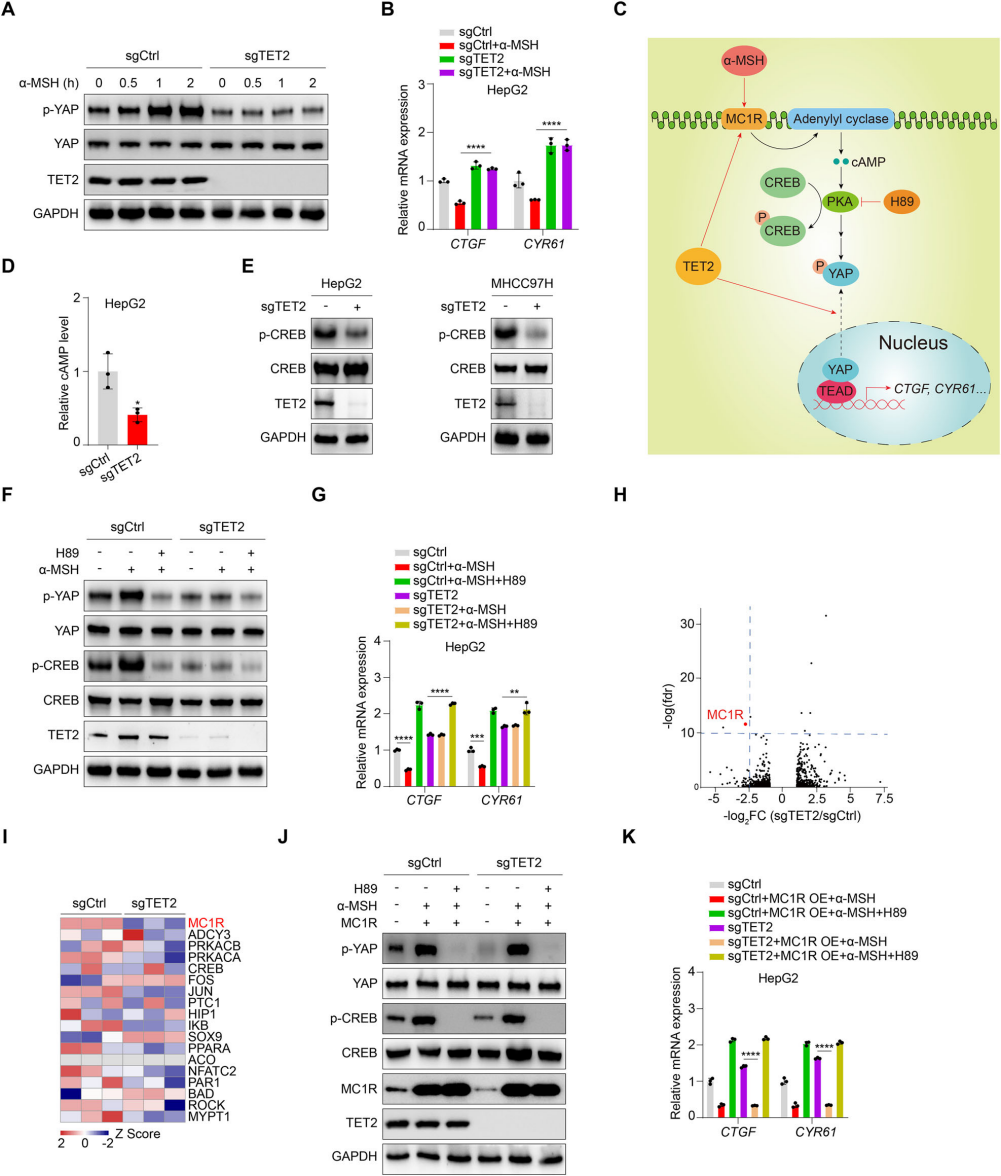
TET2 suppresses YAP activity via MC1R-cAMP-PKA signaling pathway. **A** α-MSH enhances phosphorylation level of YAP Ser127 in TET2-denpendent manner. Cells were treated with 2 μM α-MSH for different times as indicated. **B** *CTGF* and *CYR61* expression decrease upon α-MSH treatment but remain stable in sgTET2 cells. Cells were treated with 2 μM α-MSH for 2 h. **C** Schematic description of MC1R-cAMP-PKA signaling pathway. **D** Cellular cAMP concentration is decreased upon TET2 deficiency. **E** Phosphorylation of CREB Ser133 is decreased upon TET2 deficiency. **F** PKA inhibitor H89 could further lower phosphorylation level of YAP Ser127 in sgTET2 HepG2. Cells were treated with 2 μM α-MSH and/or 10 μM H89 for 1 h. **G** H89 could further stimulate expression of *CTGF* and *CYR61* in sgTET2 HepG2. Cells were treated with 2 μM α-MSH and/or 10 μM H89 for 1 h. **H** MC1R is one of the markedly downregulated genes in sgTET2 cells through RNA-seq analysis. Volcano plot showing RNA profiling for HepG2 cells deletion of TET2 compared with control cells. **I** MC1R ranked top among genes included in MC1R-cAMP-PKA signaling pathway through RNA seq analysis. **J** Overexpression of MC1R rescues phosphorylation level of YAP Ser127 in response to α-MSH upon TET2 deletion. Cells were treated with 2 μM α-MSH and/or 10 μM H89 for 1 h. **K** Overexpression of MC1R reverses expression of *CTGF* and *CYR61* in response to α-MSH upon TET2 deletion. Cells were treated with 2 μM α-MSH and/or 10 μM H89 for 1 h. Data are presented as mean ± s.d., *n* = 3 independent repeats. Unpaired, two-tailed *t*-test.

As multiple physiological signals are responsible for regulation of p-YAP level through GPCRs, various well-known extracellular cues, such as glucose or serum starvation, dihydrexidine, glucagon, epinephrine and α-MSH [22, 39, 40] were applied to figure out the exact factor mediating the alteration of p-YAP level upon TET2 deficiency. Intriguingly, we found that only α-MSH treatment could enhance p-YAP level in control cells rather than sgTET2 cells (Fig. 2A), whereas other diverse signals regulate p-YAP level independent of TET2 (Supplementary Fig. S2A–E). Consistently, YAP target gene expression declines upon α-MSH treatment in control cells but remain stable in sgTET2 cells (Fig. 2B), implying that TET2 is required for α-MSH to manipulate YAP activity (Fig. 2C). To validate the hypothesis, we found that cellular cAMP level is significantly reduced in sgTET2 cells (Fig. 2D). As an effector of cAMP, PKA activity is decreased upon TET2 deficiency as indicated by phosphorylation of CREB Ser133 (hereafter p-CREB) (Fig. 2E). Likewise, PKA inhibitor H89 [41] could reverse the change of p-YAP level and the expression of YAP target genes induced by sgTET2 (Fig. 2F, G). Surprisingly, RNA-seq analysis reveals that MC1R, the major member of melanocortin receptors, is one of the markedly downregulated genes in sgTET2 cells (Fig. 2H) and ranks top among genes involved in cAMP-PKA signaling pathway (Fig. 2I). In line with this, overexpression of MC1R in sgTET2 cells restores α-MSH-induced upregulation of p-YAP level and downregulation of YAP target gene expression (Fig. 2J, K). Taken together, these evidences demonstrate that TET2 suppresses YAP activity via MC1R-cAMP-PKA signaling pathway.

### TET2 promotes MC1R expression in HCC cells

**Fig. 3:**
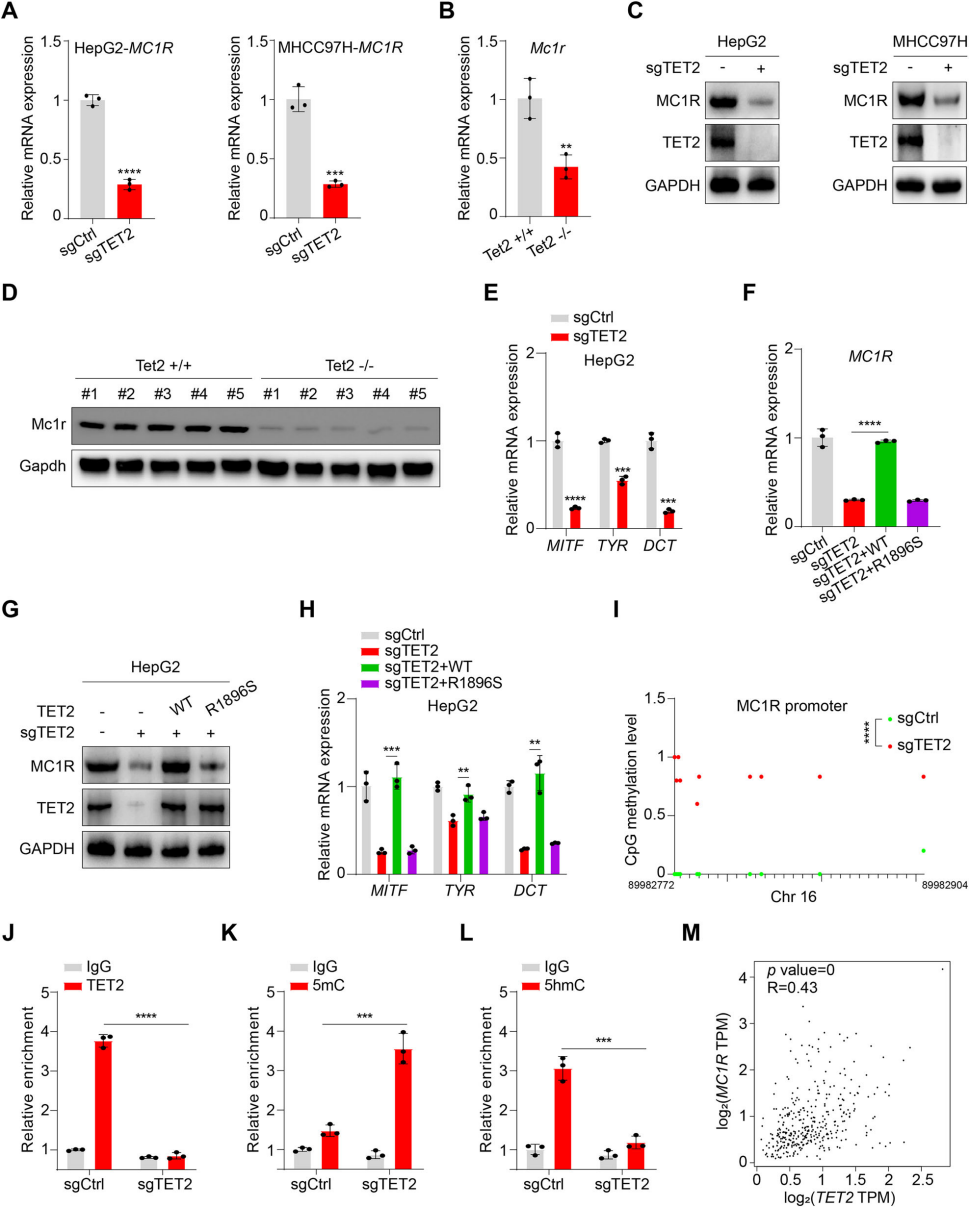
TET2 promotes MC1R expression. TET2 knockout decreases mRNA levels of *MC1R* in HCC cells (**A**) and livers of Tet2 knockout mice (**B**). TET2 knockout decreases protein levels of MC1R in HCC cells (**C**) and livers of Tet2 knockout mice (**D**). **E** Expression of MC1R downstream genes, that is, *MITF*, *TYR* and *DCT*, are evidently decreased in sgTET2 cells. Re-introduction of WT TET2 but not catalytic mutant R1896S could rescue mRNA (**F**) and protein (**G**) level of MC1R. **H** Re-introduction of WT TET2 could reverse expression of *MITF*, *TYR* and *DCT*. **I** Whole genome bisulfite sequencing analysis reveals sgTET2 cells harbors enriched CpG sites in promoter of *MC1R*. Every dot represents single CpG site. **J** ChIP-qPCR analysis shows TET2 associates promoter of *MC1R*. **K** TET2 knockout increases 5mC level of *MC1R* promoter. **L** TET2 knockout reduces 5hmC level of *MC1R* promoter. **M** *TET2* expression is positively related with *MC1R* in liver hepatocellular carcinoma. Plots show the Pearson’s correlation between *TET2* and *MC1R* mRNA level from RNA-seq data in TCGA LIHC calculated by GEPIA (Gene Expression Profiling Interactive Analysis). R and *p* values are shown. LIHC, liver hepatocellular carcinoma. Data are presented as mean ± s.d., *n* = 3 independent repeats. Unpaired, two-tailed *t*-test.

To further verify the regulatory mechanism of TET2 on MC1R-cAMP-PKA signaling, we examined MC1R expression in sgTET2 cells and livers of Tet2 knockout mice and revealed that both mRNA (Fig. 3A, B) and protein levels (Fig. 3C, D) of MC1R are declined upon TET2 deficiency. Besides, the expression of MC1R downstream genes, that is, *MITF*, *TYR* and *DCT*, are evidently decreased in sgTET2 cells (Fig. 3E). Moreover, re-introduction of WT TET2 but not catalytic mutant R1896S could rescue the expression of MC1R and its downstream genes in sgTET2 HepG2 cells (Fig. 3F–H). Given the role of TET2 on DNA demethylation, we carried out whole genome bisulfite sequencing analysis to strengthen the molecular mechanism of TET2-mediated MC1R expression. Surprisingly, the methylation level of multiple CpGs located in MC1R promoter region is distinctly increased in sgTET2 cells (Fig. 3I), which evokes us to further inspect whether TET2 binds to the promoter of MC1R. We conducted ChIP-qPCR analysis and found that TET2 could bind to the promoter of MC1R (Fig. 3J), decrease the 5mC level (Fig. 3K) and increase the 5hmC level (Fig. 3L). Notably, the mRNA level of TET2 is positively related with MC1R in HCC (Fig. 3M), providing clinical evidence for TET2 regulation of MC1R. Together, TET2 enhances MC1R expression by oxidation of 5mC in MC1R promoter.

### α-MSH is non-essential for MC1R to repress YAP activity

**Fig. 4:**
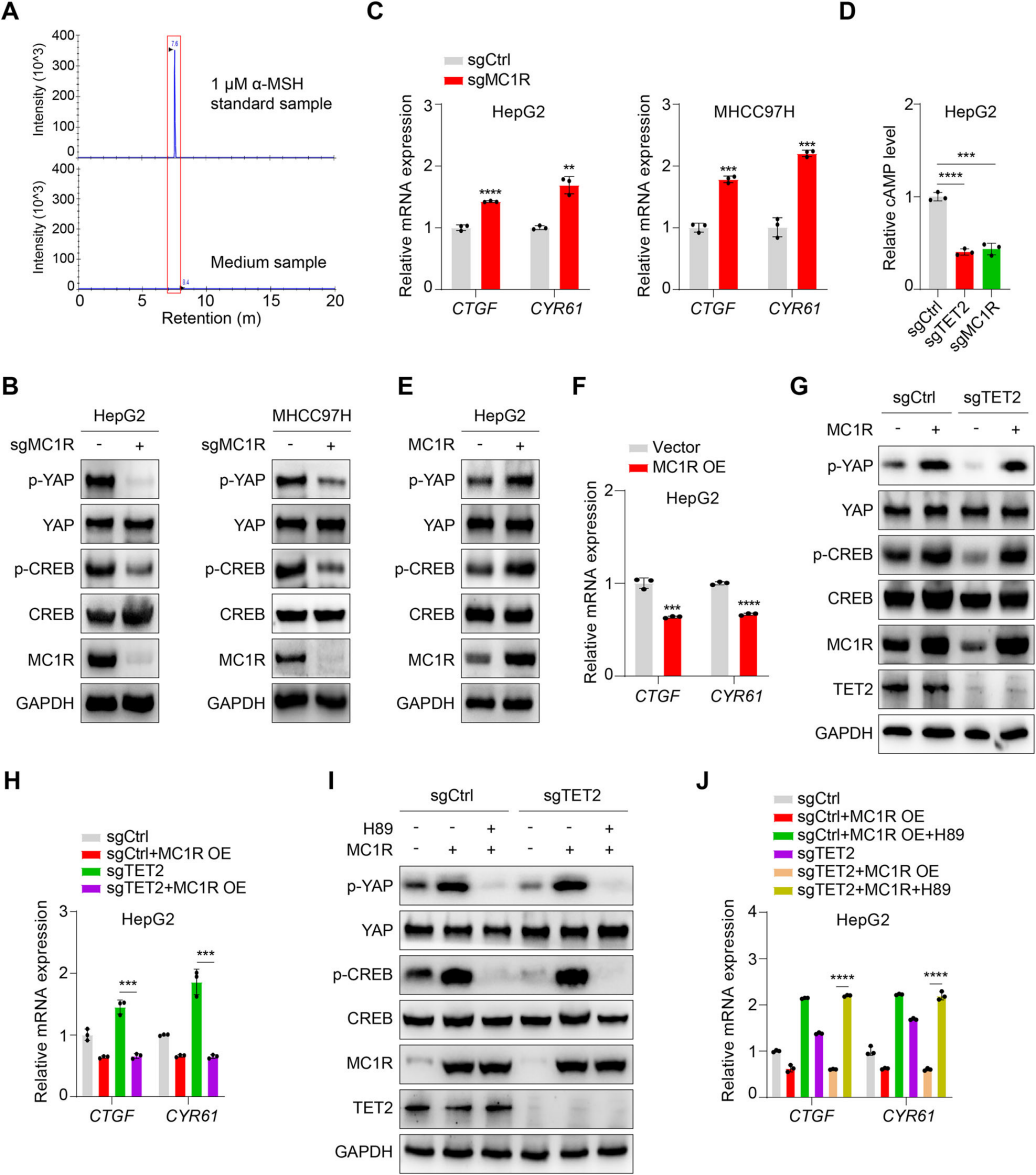
MC1R alone can repress YAP activity independent of its ligand. **A** α-MSH concentration in the complete DMEM medium was measured by LC-MS with 1 μM α-MSH as a standard sample. Data are shown as intensity. **B** Phosphorylation level of YAP Ser127 is significantly decreased upon deletion of MC1R in HCC cells. **C** *CTGF* and *CYR61* are distinctly upregulated in sgMC1R HCC cells. **D** Cellular cAMP concentration is decreased upon MC1R deficiency. **E** Overexpression of MC1R induce upregulated phosphorylation level of YAP Ser127. **F** Overexpression of MC1R induce downregulated expression of *CTGF* and *CYR61*. **G** Re-introduce MC1R into sgTET2 HepG2 could rescue phosphorylation level of YAP Ser127. **H** Overexpression of MC1R compromise expression of *CTGF* and *CYR61* in sgTET2 HepG2. **I** MC1R alone can promote phosphorylation level of YAP Ser127 in sgTET2 HepG2 independent of α-MSH. **J** MC1R alone can suppress expression of *CTGF* and *CYR61* in sgTET2 HepG2 independent of α-MSH. Data are presented as mean ± s.d., *n* = 3 independent repeats. Unpaired, two-tailed *t*-test.

Given that these experiments above were conducted without α-MSH treatment, we tried to measure α-MSH concentration in the complete DMEM medium with 10% FBS by LC-MS. Surprisingly, α-MSH is undetectable in the medium (Fig. 4A). Therefore, we speculate that α-MSH is non-essential for MC1R to repress YAP activity, since previous study has pinpointed that activation of MC1R signaling could be independent of its agonist [42]. Knockout of MC1R (hereafter sgMC1R) significantly impairs PKA and YAP activity as indicated by p-CREB and p-YAP levels, respectively (Fig. 4B), thereby enhancing the expression of *CTGF* and *CYR61* (Fig. 4C). Moreover, deficiency of MC1R mimics the effect of TET2 knockout on cAMP-PKA pathway as indicated by the reduced level of cAMP (Fig. 4D). Consistently, overexpression of MC1R not only induces the upregulation p-YAP level (Fig. 4E) and downregulation of its target gene expression (Fig. 4F) but also reverse the change upon deficiency of TET2 (Fig. 4G, H). Moreover, PKA inhibitor H89 prominently blocks MC1R-mediated repression of YAP (Fig. 4I) and expression of its target genes (Fig. 4J). Collectively, these findings demonstrate MC1R modulates YAP activity regardless of α-MSH.

### TET2-MC1R-YAP axis is crucial for tumor growth

**Fig. 5:**
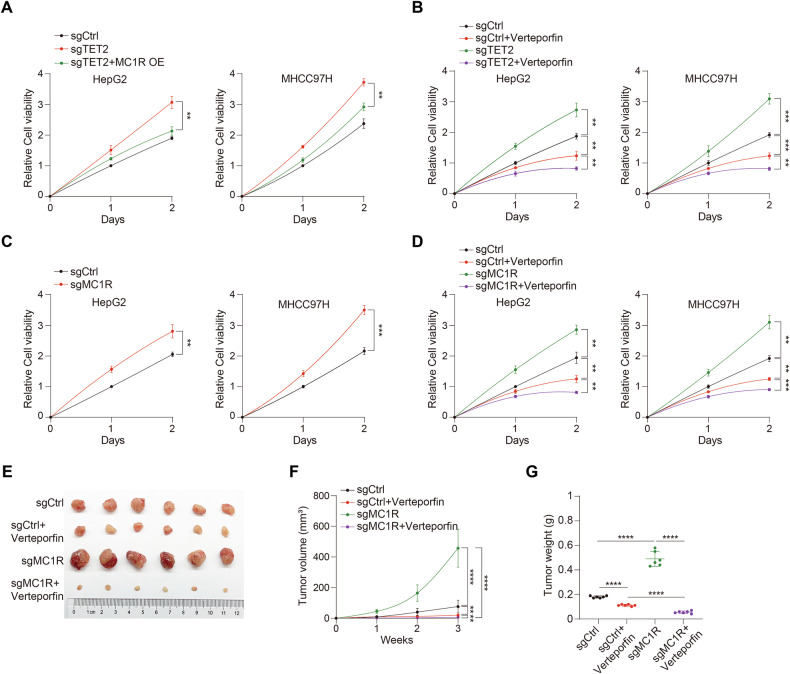
TET2-MC1R-YAP axis is crucial for tumor growth. **A** Introduction of MC1R could reverse the growth advantage induced by lack of TET2. **B** Verteporfin dramatically deteriorates the growth advantage of sgTET2 cells. HCC cells were treated with 1 μM verteporfin for different times as indicated. **C** MC1R knockout promotes the proliferation of HCC cells. **D** Verteporfin disrupts the growth advantage of sgMC1R cells. HCC cells were treated with 1 μM verteporfin for different times as indicated. **E**–**G** Verteporfin abrogates the growth advantage aroused by deficiency of MC1R in vivo. Tumor image (**E**), tumor volume (**F**) and tumor weight (**G**) were monitored with three weeks after transplantation. *n* = 6 independent animals. Data are presented as mean ± s.d., *n* = 3 independent repeats. Unpaired, two-tailed *t*-test.

To determine the functional significance of TET2-MC1R-YAP axis, we examined the cell proliferation regulated by the axis and found that deficiency of TET2 facilitates HCC cell proliferation, while introduction of MC1R could impair the proliferation advantage induced by TET2 knockout (Fig. 5A). Verteporfin dramatically suppresses the proliferation of sgTET2 cells (Fig. 5B). Next, we tried to uncover the functional role of MC1R in cancer cell proliferation and tumor growth. Results showed that knockout of MC1R accelerates the cell proliferation (Fig. 5C), and this effect could be reversed by verteporfin treatment as well (Fig. 5D). To further valid the suppressive function of MC1R in vivo, we carried out xenograft model by subcutaneously injecting sgMC1R HCC cells into athymic nude mice and monitored the tumor growth. Consequently, lack of MC1R boosts tumor growth, which can be dramatically repressed by verteporfin treatment (Fig. 5E–G). These results demonstrates that MC1R functions as a tumor suppressor of HCC by suppressing YAP activity.

### Activation of MC1R signaling dampens HCC growth

**Fig. 6:**
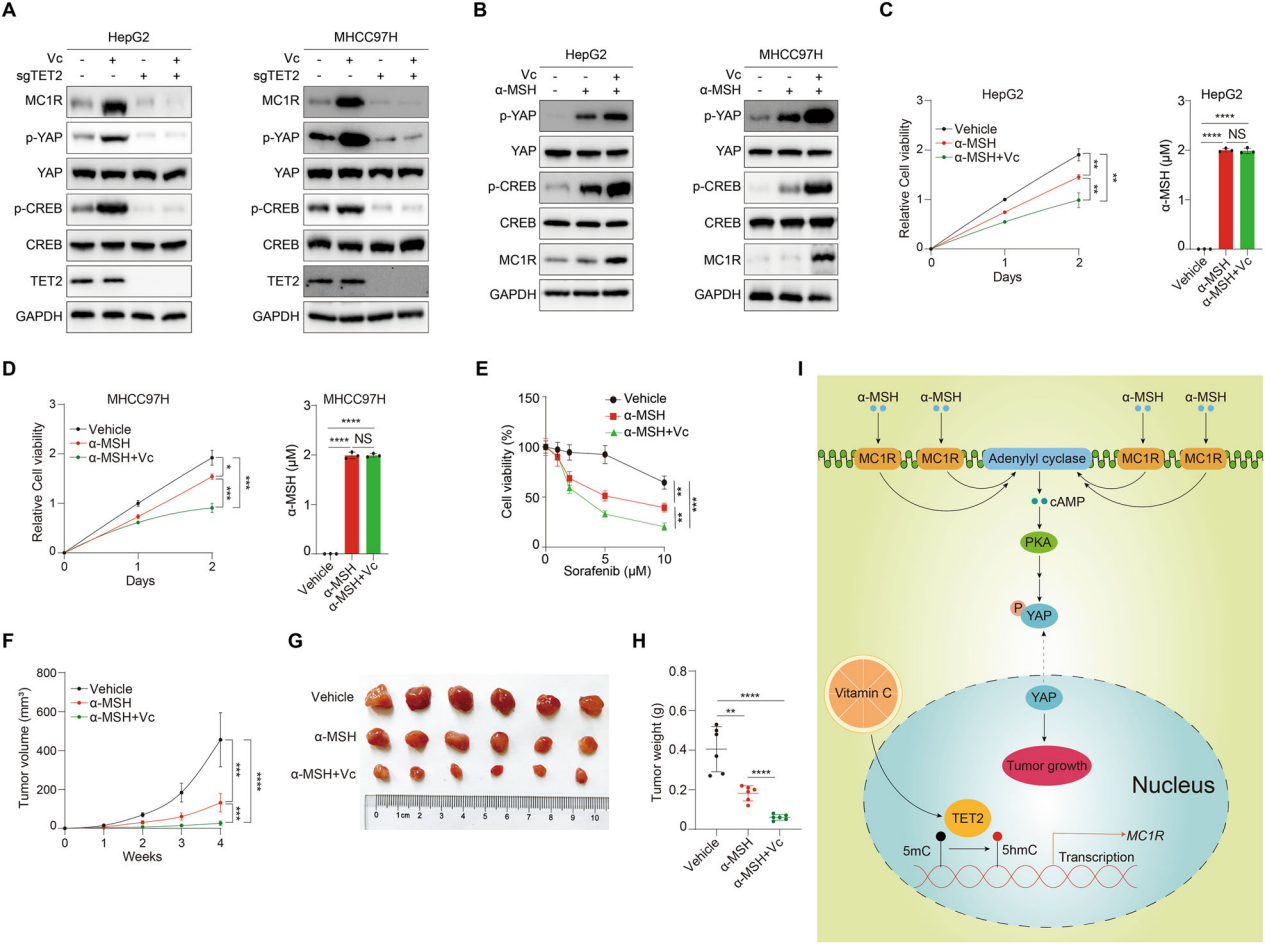
Activation of MC1R signaling dampens HCC growth. **A** Vitamin C promotes the expression of MC1R and phosphorylation level of YAP Ser127 via activation of TET2 in HCC cells. Cells were treated with 1 mM vitamin C for 24 h. **B** Vitamin C can enhance the suppressive effect of α-MSH on YAP activity in HCC cells. Cells were treated with 1 mM vitamin C for 24 h and 2 μM α-MSH for 1 h. **C**, **D** Vitamin C can enhance the anticancer effect of α-MSH in HCC cells. Cells were treated with 2 μM α-MSH alone or combination of 1 mM vitamin C and 2 μM α-MSH for different times as indicated (left panel). α-MSH concentration was measured (right panel). **E** Vitamin C and α-MSH can overcome sorafenib resistance in sorafenib-resistant MHCC97H cells. Cells were treated with 2 μM α-MSH alone or combination of 1 mM vitamin C and 2 μM α-MSH for three days. Vitamin C enhances the anticancer effect of α-MSH in vivo. Tumor image (**F**), tumor volume (**G**) and tumor weight (**H**) were monitored with four weeks after transplantation. *n* = 6 independent animals. **I** TET2 stimulates transcription of MC1R via oxidation of 5mC involved in its promoter, which can be enhanced by vitamin C. Afterwards, MC1R boosts cAMP-PKA signaling in both ligand dependent and independent manner to repress YAP activity. Combination of vitamin C and α-MSH can distinctly dampen tumor growth. Data are presented as mean ± s.d., *n* = 3 independent repeats. Unpaired, two-tailed *t*-test.

Since MC1R functions as a tumor suppressor in HCC, we attempted to propose a novel strategy for HCC therapy via activation of MC1R signaling. Vitamin C has been elucidated as a compound with anticancer properties through multiple mechanisms, among which TET2 is an important target of vitamin C. Vitamin C not only acts as a cofactor of TET2, but also enhances TET2 activity as a DNA dioxygenase, all of which raise the notion that vitamin C functions as an activator of TET2 [15, 43]. Hence, we employed vitamin C to investigate its effect on TET2-MC1R-YAP axis. Consequently, both MC1R expression and p-YAP levels are remarkably increased upon vitamin C treatment, while deficiency of TET2 compromises the effect (Fig. 6A). In line with the suppressive role of MC1R signaling in tumor growth, we initiated the hypothesis that vitamin C might enhance the anticancer effect of α-MSH. Compared with α-MSH alone, combination of α-MSH and vitamin C significantly promotes p-YAP level (Fig. 6B) and suppresses cell proliferation (Fig. 6C, D). Interestingly, activation of MC1R signaling could overcome sorafenib resistance of HCC cells (Fig. 6E). Furthermore, we proposed vitamin C synergizes with α-MSH treatment and the combination therapy dramatically represses tumor growth in vivo (Fig. 6F–H). Together, by stimulating the expression of MC1R accompanied with its ligand α-MSH treatment, MC1R signaling is further amplified to dampen tumor growth (Fig. 6I), proposing targetable vulnerability for HCC therapy.

### TET2-MC1R-YAP axis correlates with sorafenib sensitivity and prognosis of HCC

**Fig. 7:**
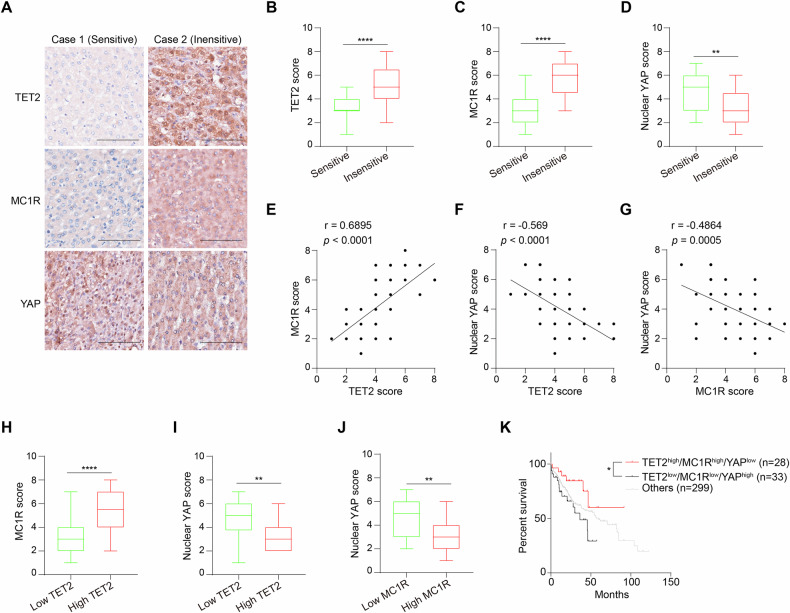
TET2-MC1R-YAP axis correlates with sorafenib sensitivity and prognosis for HCC. **A** Immunohistochemical staining with anti-TET2, anti-MC1R and anti-YAP antibodies was performed on 48 LIHC specimens receiving sorafenib therapy. Representative images are shown. Scale bars, 100 μm. TET2 (**B**) and MC1R (**C**) are highly expressed in sorafenib-insensitive LIHC specimens. **D** Nuclear YAP is highly expressed in sorafenib-sensitive LIHC specimens. **E**–**G** Pearson correlation test was used to analyze the relationship between the score of TET2 and MC1R (**E**), the score of TET2 and nuclear YAP (**F**), and the score of MC1R and nuclear YAP (**G**). Note that certain dots on the graphs represent more than one specimen and are shown as overlapping. **H** MC1R is highly expressed in LIHC patients with high TET2 expression. Nuclear YAP is highly expressed in LIHC patients with low TET2 expression (**I**) or low MC1R expression (**J**). **K** TET2-MC1R-YAP axis harbors potential significance for prognosis of LIHC. The combination of TET2, MC1R and YAP expression is a potential marker for prognosis of LIHC. 360 LIHC samples from TCGA database were divided into three groups based on the expression of TET2, MC1R and YAP and the prognosis of these groups was analyzed.

The clinical relevance of TET2-MC1R-YAP axis was further validated by immunohistochemical analysis from 48 LIHC specimens receiving sorafenib therapy (Fig. 7A and Supplementary Table 1). Results showed that both the expression of TET2 and MC1R negatively associates with the sensitivity to sorafenib (Fig. 7B, C), while the level of nuclear YAP is positively associated with the sensitivity to sorafenib (Fig. 7D). In line with physiological influence, the level of TET2 positively correlates with MC1R level (Fig. 7E). Nevertheless, both TET2 and MC1R levels negatively correlate to the level of nuclear YAP (Fig. 7F, G). Patients with high TET2 expression levels consistently recapitulate higher MC1R and lower nuclear YAP score (Fig. 7H, I). Moreover, patients harboring high MC1R expression present low nuclear YAP score (Fig. 7J). Eventually, to explore the prognostic significance of TET2-MC1R-YAP axis, we classified LIHC samples from TCGA database into three groups based on the expression levels of TET2, MC1R and YAP, that is, TET2^high^/MC1R^high^/YAP^low^, TET2^low^/MC1R^low^/YAP^high^ and others. Overall survival analysis uncovers that the TET2^low^/MC1R^low^/YAP^high^ group presents worse outcome in LIHC compared with TET2^high^/MC1R^high^/YAP^low^ group (Fig. 7K), implying the potential significance of TET2-MC1R-YAP axis for prognosis of LIHC. Taken together, these findings not only enlighten the prominence of TET2-MC1R-YAP axis in HCC growth and sensitivity to sorafenib, but also identify its clinical relevance for prognosis of HCC.

## Discussion

Here we report that DNA methylcytosine dioxygenase TET2 catalyzes demethylation of MC1R promoter to facilitate its transcription. MC1R further triggers phosphorylation and inactivation of YAP via cAMP-PKA signaling to restrain tumor growth. Physiologically, TET2-MC1R-YAP axis modulates cellular resistance to sorafenib and acts as a potential biomarker for prognosis of HCC. Combination of TET2 activator vitamin C and MC1R ligand α-MSH significantly dampens tumor growth (Fig. 6I). Collectively, this study not only clarifies the molecular link between epigenetic dependency and drug resistance, but also proposes a novel GPCR-targeted strategy for HCC therapy.

The majority of HCC patients are diagnosed with advanced stage and unable to receive ablation, resection and transplantation. Sorafenib, lenvatinib and regorafenib are conventional targeted drugs for advanced HCC [2]. Unfortunately, patients always acquire drug resistance within several months, giving prominence to the demand for original strategies to alleviate drug resistance. Besides loss-of-function mutations, TET2 activity is differentially fine-tuned in multiple solid tumors. For instance, tumors harboring IDH1/2 mutation are characterized by production of 2-hydroxyglutarate, a competitive inhibitor of α-KG-dependent dioxygenases, including TET2 [44, 45]. Moreover, various cofactors are involved in the regulation of TET2 activity, such as Fe^2+^ and oxygen [12], implying broad clinical potential of diverse therapies based on the activity or expression of TET2. Therefore, our findings initiate stratification strategies of sorafenib for patients with TET2 loss or inactivation.

α-MSH has been revealed to convey proliferation and invasion blockade of melanoma [46, 47]. Consistently, activation of MC1R could prevent melanomagenesis [48]. In our model, combination of α-MSH and vitamin C or α-MSH alone exerts suppressive effect on HCC growth. Nevertheless, α-MSH enables to nurture immune surveillance evasion of melanoma via a bypass effect [49]. Pituitary derived α-MSH was reported to promote myelopoiesis and immunosuppression to accelerate tumor growth via activating MC5R, since MC5R is highly expressed in bone marrow progenitors [50]. Therefore, further investigations of α-MSH and its receptors are needed to stratify the limitations clinically. Vitamin C has been demonstrated to repress tumor growth in multiple studies, whereas the clinical potential of vitamin C as an anticancer compound may also lie in its combined utilization with other therapies. Intravenous vitamin C combined with chemotherapy, radiation therapy or targeted therapy are undergoing various clinical trials as yet [43]. Here we introduce an innovative strategy for the management of HCC by combining vitamin C with α-MSH. This study not only broadens the scope of vitamin C application, but also puts forward potential implications for HCC therapy.

## Methods

### Cell culture and transfection

HepG2 and 293T were obtained from National Collection of Authenticated Cell Cultures. MHCC97H was obtained from Liver Cancer Institute of Fudan University. All cell lines have been authenticated by STR fingerprinting and tested for mycoplasma contamination prior to experiments. Cells were cultured in DMEM (Meilun Biotechnology, Dalian, China) supplemented with 1× antibiotics (containing Penicillin-Streptomycin-Amphotericin B) and 10% FBS (Biological Industries, Israel). Plasmids were transfected into cells with EZ Trans (Life-iLab, Shanghai, China) following the instructions. The Cell Counting Kit-8 (New Cell & Molecular Biotech, China) was used to monitor cell proliferation according to the manufacturer’s protocol. Sorafenib, lenvatinib, regorafenib, α-MSH, epinephrine and glucagon were purchased from Meilun Biotechnology (Dalian, China). Verteporfin was purchased from MedChemExpress (Shanghai, China). H89 and dihydrexidine were purchased from TargetMol (USA). Vitamin C was purchased from Yeasen (Shanghai, China).

### Antibodies

TET2 (Cat. #18950S, 1:1000 dilution) and PARP (Cat. #9542T, 1:1000 dilution) antibodies were purchased from Cell Signaling Technology. GAPDH (Cat. #60004-1-Ig, 1:10000 dilution) and TUBULIN (Cat. #11224-1-AP, 1:10000 dilution) antibodies were purchased from Proteintech. The following antibodies were purchased from HUABIO and used at the indicated dilutions for western blot analysis and immunohistochemistry: YAP (Cat. #ET1608-30, 1:1000 dilution), p-YAP (Ser 127) (Cat. #ET1611-69, 1:1000 dilution), MC1R (Cat. #ER63924, 1:1000 dilution), CREB (Cat. #ET1601-15, 1:500 dilution), p-CREB (Ser 133) (Cat. #ET7107-93, 1:500 dilution).

### CRISPR-mediated gene deletion

sgRNAs were cloned into pLentiCRIPSR v2 vector. CRISPR-Cas9 lentivirus is produced by transfecting 6 μg sgRNA plasmid, 4.5 μg psPAX2 and 1.5 μg pMD2.G into HEK-293T cells in 100 mm dishes. Supernatant was collected at 48 h and 72 h after transfection and stored in -80 °C for infection. The sgRNA sequences targeting individual genes were as follows: TET2#1 (GATTCCGCTTGGTGAAAACG), MC1R#1 (CATCGCCTACTACGACCACG), MC1R#2 (CGTTGCTCCCGCTCACCAGC).

### Western blot and immunofluorescence staining

Cells or tissues were washed with PBS for three times, followed by treatment with 0.5% NP-40 lysis buffer containing protease inhibitor cocktail (APExBIO, Houston, USA). For phosphorylation sample preparation, 1× phosphatase inhibitor cocktail (TargetMol, USA) was added into the lysate. EZ Protein any KD PAGE kit (Life-iLab, Shanghai, China) was applied for SDS-PAGE electrophoresis. For liver tissue phosphorylation sample, 4–12% Precast Bis-Tris Gel (Tanon, Shanghai, China) was used.

For immunofluorescence staining, HepG2 cells were seeded onto coverslips at 50% confluency prior to experiment. Cells were fixed with 4% paraformaldehyde for 15 min followed by incubation with 0.3% Triton X-100 at room temperature for 15 min. Then cells were blocked with goat serum at room temperature for 30 min. After that, the slides were incubated with YAP primary antibody at 4 °C overnight. After washing with PBS, the slides were incubated with secondary antibody at room temperature for 30 min. The slides were mounted with antifade reagent and samples were observed using confocal laser scanning microscope (Nikon).

### RNA extraction, qPCR and RNA-seq analysis

The total RNA was extracted using EZ-press RNA purification kit (EZ Bioscience, USA). 1 μg total RNA was reverse-transcribed by EZ Bioscience-RT mix (EZ Bioscience, USA). qPCR was performed using SYBR qPCR Master Mix (Yeasen, Shanghai, China). All primers were synthesized by BioSune (Shanghai, China). RNA-seq analysis was conducted by Origin-gene (Shanghai, China).

The primers used (forward and reverse, respectively) were as follows: human *GAPDH* (GTCTCCTCTGACTTCAACAGCG and ACCACCCTGTTGCTGTAGCCAA), human *MC1R* (ACCTGCACTCGCCCATGTATTACT and AATGATGCCCAGGAAGCAGAGACT), human *CTGF* (CCAATGACAACGCCTCCTG and TGGTGCAGCCAGAAAGCTC), human *CYR61* (AGCCTCGCATCCTATACAACC and TTCTTTCACAAGGCGGCACTC), human *MITF* (CAGTCCGAATCGGGGATCG and TGCTCTTCAGCGGTTGACTTT), human *TYR* (TGCACAGAGAGACGACTCTTG and GAGCTGATGGTATGCTTTGCTAA), human *DCT* (CTTGGGCTGCAAAATCCTGC and CAGCACTCCTTGTTCACTAGG), mouse *Gapdh* (AGGTCGGTGTGAACGGATTTG and GGGGTCGTTGATGGCAACA), mouse *Mc1r* (GTGCTGGTTGTGATAGCCATC and TGCTGACACTTACCATCAGGT), mouse *Ctgf* (GGCCTCTTCTGCGATTTCG and GCAGCTTGACCCTTCTCGG), mouse *Cyr61* (TAAGGTCTGCGCTAAACAACTC and CAGATCCCTTTCAGAGCGGT).

### Whole genome bisulfite sequencing analysis

Whole genome bisulfite sequencing and data analysis were conducted by Acegen (Shenzhen, China).

### Chromatin immunoprecipitation assay

The Chromatin immunoprecipitations (ChIP) assay was carried out according to a ChIP assay kit (Cell Signaling Technology#56383). The primers used (forward and reverse, respectively) were as follows: MC1R (CCTCTCGCAGCCCTCG and GTACACACATACATACGTGCGC).

### Animal work

Tet2 knockout C57BL/6 mice (*n* = 5 independent animals) were purchased from Jackson Laboratories (USA). For transplantation of MHCC97H to BALB/c nude mice (*n* = 6 independent animals), cells were concentrated to 10^6^ per 100 μl in PBS, then subcutaneously injected into the flank of 6-week-old male BALB/c nude mouse. Sorafenib (30 mg/kg, orally), verteporfin (100 mg/kg, intraperitoneally), α-MSH (0.5 mg/kg, intraperitoneally) and vitamin C (4 g/kg, intraperitoneally) were administered every other day starting at day 4 (the day of transplantation). For subcutaneous tumor growth, tumor size must not exceed 15 mm at the largest diameter, according to the guidelines provided by the animal care program, and no experiments in this study generated a tumor burden over this limit. Tumor volume was calculated as volume = width^2^ × length × 0.5.

All animal experiments were performed as protocol approved by the ethics committee of School of Basic Medical Sciences, Fudan University. The animals’ care was in accordance with institutional guidelines.

### Immunohistochemical analysis

48 paraffin-embedded sections of LIHC tissues were carried out for immunohistochemical analysis. Tissues sections were deparaffinized and rehydrated followed with antigen retrieval by boiling sections in 10 mM citrate buffer (pH 6.0) for 5 min. 3% hydrogen peroxide was used for blockade of endogenous peroxidase. Slides were incubated with 10% FBS to remove non-specific binding sites. Sections were subsequently incubated with antibodies against TET2, MC1R and YAP. After incubation with secondary antibodies, sections were prepared for DAB and haematoxylin staining. Immunoreactivity was semiquantitatively assessed base on intensity and area: the percentage of immunoreactivity was graded on a range from 0 to 5 (Score 0 if 0% of tumor cells presented positive staining, 1 if 0–1%, 2 if 2–10%, 3 if 11–30%, 4 if 31–70% and 5 if 71–100%). The staining intensity was scored from 0 to 3 (0 if negative, 1 if weak, 2 if moderate, 3 if strong). The proportion and intensity scores were combined to calculate a total score (range: 1–8). Written informed consent was acquired and the investigation was approved by institutional review board of Shanghai Changzheng Hospital.

### TCGA database analysis

Raw RNA-seq data for LIHC tumor tissues were downloaded from TCGA database. Raw data of each sequenced gene were rescaled to set the median equal to 1. The tumor tissues were divided into two groups according to the median value of *TET2*. YAP activities were quantified by averaging the normalized expression of 20 YAP target genes, that is, *COX8A*, *CYR61*, *GGH*, *GADD45B*, *CDIPT*, *PSAT1*, *HEXB*, *GPT2*, *ITGB5*, *CTGF*, *TRIB3*, *PHGDH*, *EMP2*, *PTPMT1*, *ITGB2*, *RHOQ*, *FAM45A*, *PDLIM2, BCAT1* and *KCNK12*.

### Statistics and reproducibility

All quantitative data were presented as the mean ± standard deviation (SD) and analyzed by GraphPad Prism 8 software using two-sided unpaired Student’s *t* test. *, **, *** and **** represent *p* values of less than 0.05, 0.01, 0.001 and <0.001, respectively. All experiments were repeated independently with similar results.

## Supplementary information

Supplemental Material

Uncropped blots

## Data Availability

RNA-seq and whole genome bisulfite sequencing data for this project are available at the NCBI SRA database under accession number PRJNA781771 and PRJNA782072, respectively. All other data supporting the findings of this study are available from the corresponding author upon request.
