# Supplementary material for: TET2 orchestrates YAP signaling to potentiate targetable vulnerability in hepatocellular carcinoma
Source: Cell Death Dis. 2025 Jun 5;16(1):438. doi: 10.1038/s41419-025-07745-3 (PMC12141445; doi:10.1038/s41419-025-07745-3)
Supplement: Supplementary file 1 — Supplemental Material [file 41419_2025_7745_MOESM1_ESM.pdf]

Figure S1. Lenvatinib and regorafenib suppress cell viability and induce apoptosis independent of TET2. A, TET2 knockout promotes the proliferation of HCC cells. Cell proliferation is quantified by CCK8 assay. **B, C,** Lenvatinib suppresses cell viability and induces apoptosis independent of TET2. Cells were treated with different concentration of lenvatinib as indicated for two days (B), or 10 μM lenvatinib for different times as indicated (C). Cell viability was analyzed in top panels. Western blot analysis of cleaved PARP was performed in bottom panels. **D, E,** Regorafenib suppresses cell viability and induces apoptosis independent of TET2. Cells were treated with different concentration of regorafenib as indicated for two days (D), or 10 μM lenvatinib for different times as indicated (E). **F,** Sorafenib resistant cells exhibit higher protein level of YAP. Cell viability was analyzed in left panel. Western blot analysis of YAP was performed in right panels. **G,** TET2 knockout fails to rescue sorafenib sensitivity in sorafenib resistant MHCC97H. Cell viability was analyzed in left panels. Western blot analysis of TET2 was performed in right panels. Data are presented as mean ± s.d., n = 3 independent repeats. Unpaired, two-tailed t-test.

Figure S2. A multitude of intrinsic and extrinsic signals enhance phosphorylation level of YAP Ser127 independent of TET2. A, Glucose starvation enhances phosphorylation level of YAP Ser127 independent of TET2. Cells were deprived of glucose for 12 h. **B,** Serum starvation enhances phosphorylation level of YAP Ser127 independent of TET2. Cells were deprived of serum for different times as indicated. **C,** Dihydrexidine enhances phosphorylation level of YAP Ser127 independent of TET2. Cells were treated with 10 μM dihydrexidine for different times as indicated. **D,** Glucagon enhances phosphorylation level of YAP Ser127 independent of TET2. Cells were treated with 2 μM glucagon for different times as indicated. **E,** Epinephrine enhances phosphorylation level of YAP Ser127 independent of TET2. Cells were treated with 10 μM epinephrine for different times as indicated. Data are presented as mean ± s.d., n = 3 independent repeats. Unpaired, two-tailed t-test.

Figure S1

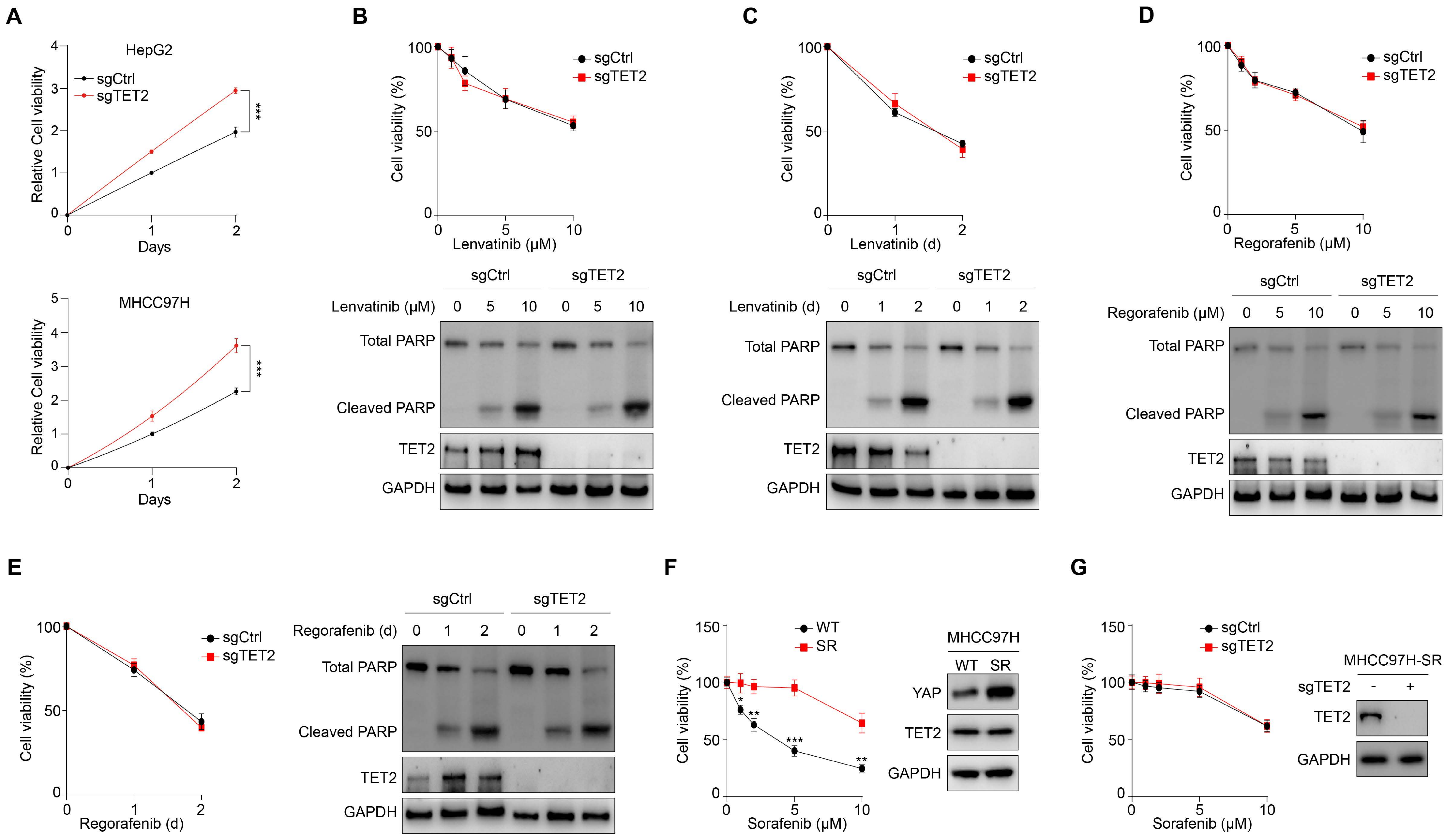
A
HepG2
sgCtrl
sgTET2
Relative Cell viability
Days
***
MHCC97H
B
Cell viability (%)
Lenvatinib (μM)
sgCtrl
sgTET2
Lenvatinib (μM) 0 5 10 0 5 10
Total PARP
Cleaved PARP
TET2
GAPDH
C
Lenvatinib (d)
Lenvatinib (d) 0 1 2 0 1 2
D
Regorafenib (μM)
Regorafenib (μM) 0 5 10 0 5 10
E
Regorafenib (d)
Regorafenib (d) 0 1 2 0 1 2
F
WT
SR
Sorafenib (μM)
MHCC97H
YAP
G
MHCC97H-SR
sgTET2 - +

Figure S2

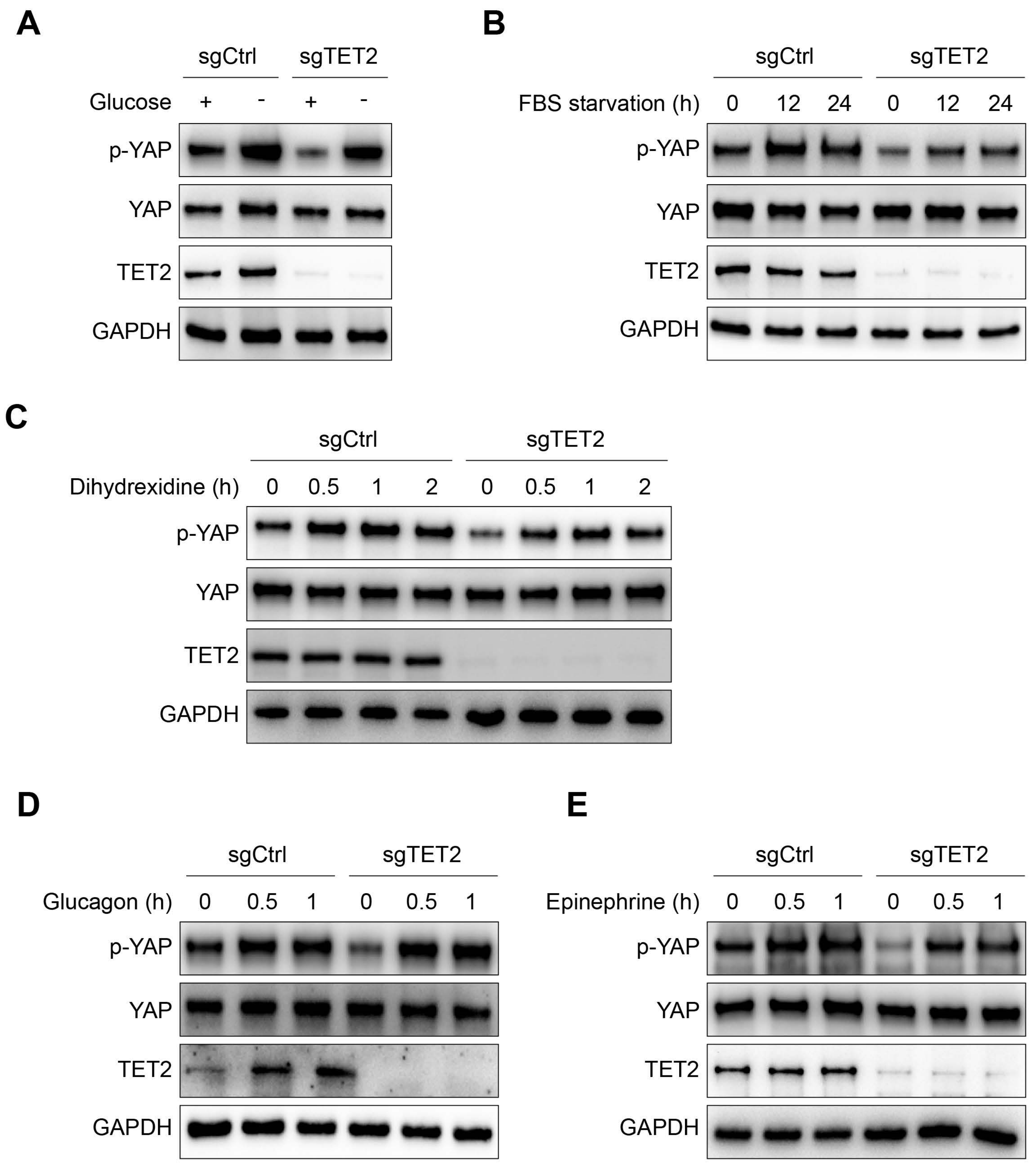

A
sgCtrl
sgTET2
Glucose
+
-
+
-
p-YAP
YAP
TET2
GAPDH
B
sgCtrl
sgTET2
FBS starvation (h)
0
12
24
0
12
24
p-YAP
YAP
TET2
GAPDH
C
sgCtrl
sgTET2
Dihydrexidine (h)
0
0.5
1
2
0
0.5
1
2
p-YAP
YAP
TET2
GAPDH
D
sgCtrl
sgTET2
Glucagon (h)
0
0.5
1
0
0.5
1
p-YAP
YAP
TET2
GAPDH
E
sgCtrl
sgTET2
Epinephrine (h)
0
0.5
1
0
0.5
1
p-YAP
YAP
TET2
GAPDH

Supplementary Table 1

Demographic and clinical characteristics of patients		
**Clinicopathological parameters**	**Sorafenib responder (n = 31)**	**Sorafenib non-responder (n = 17)**
**Age(year)**		
<55	13	8
≥55	18	9
**Gender**		
Male	28	14
Female	3	3
**Liver cirrhosis**		
No	24	15
Yes	7	2
**Neoadjuvant therapy**		
No	9	5
Yes	22	12
**Barcelona-Clinic liver cancer stage (BCLC)**		
0/A	23	12
B	3	4
C	2	0
D	3	1
**Tumor number**		
<3	29	15
≥3	2	2
**Tumor size(cm)**		
≤5	19	7
>5	12	10
**TNM stage**		
I/II	25	14
III/IV	6	3
**Microvascular invasion (MVI)**		
M0	19	9
M1	8	6
M2	4	2
**Edmondson-Steiner grade**		
I/I-II	9	6
II/II-III	15	7
III/III-IV	7	4
**Ki67(%)**		
<50	28	13
≥50	3	4
**Extrahepatic spread**		
No	25	9
Yes	6	8
**Intrahepatic tumor recurrence**		
No	20	5
Yes	11	12
