# Supplementary figures and images for: TET2 orchestrates YAP signaling to potentiate targetable vulnerability in hepatocellular carcinoma
Source: Cell Death Dis. 2025 Jun 5;16(1):438. doi: 10.1038/s41419-025-07745-3 (PMC12141445; doi:10.1038/s41419-025-07745-3)

**Figure 1**

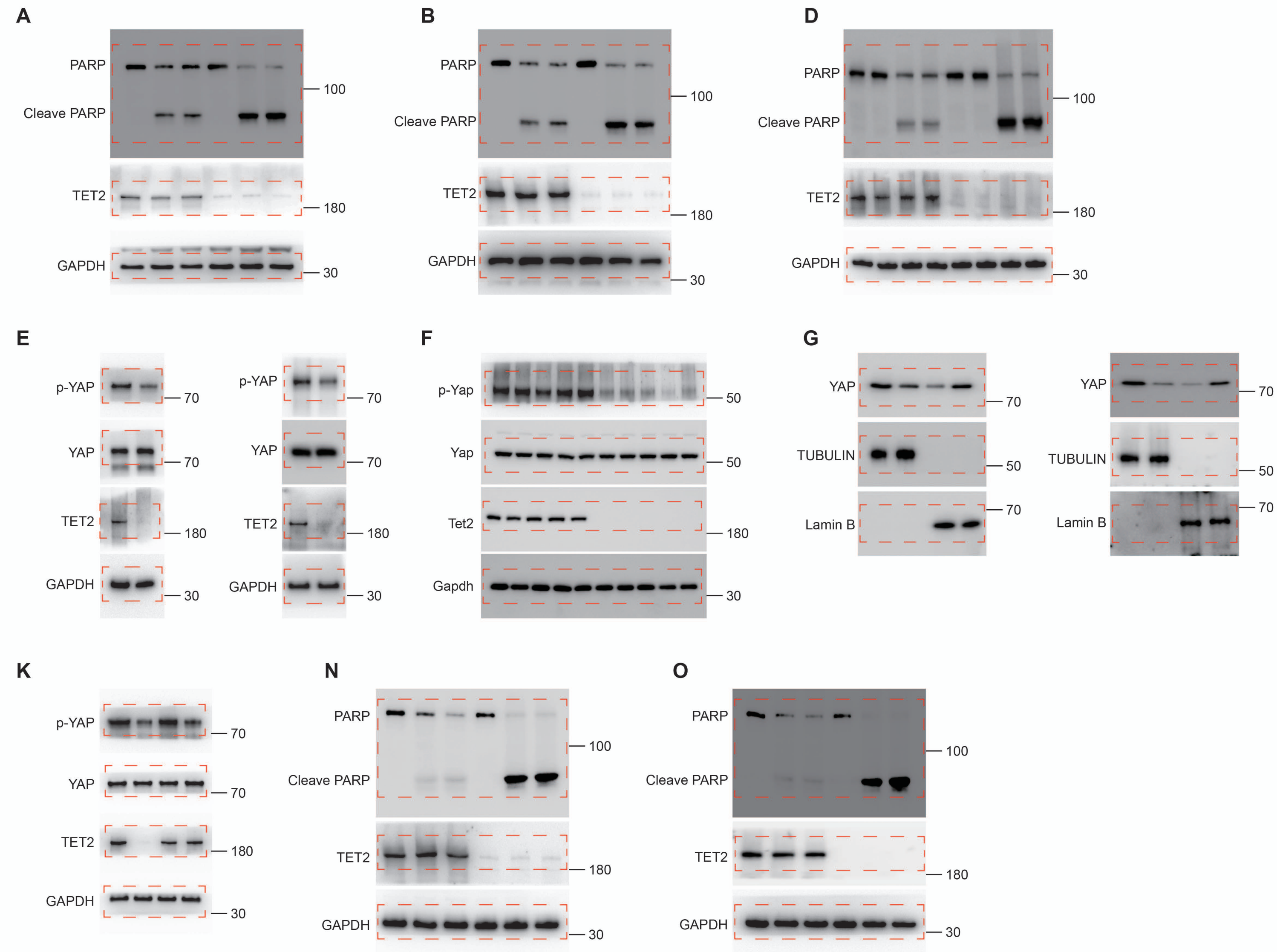

**Figure 2**

**A**

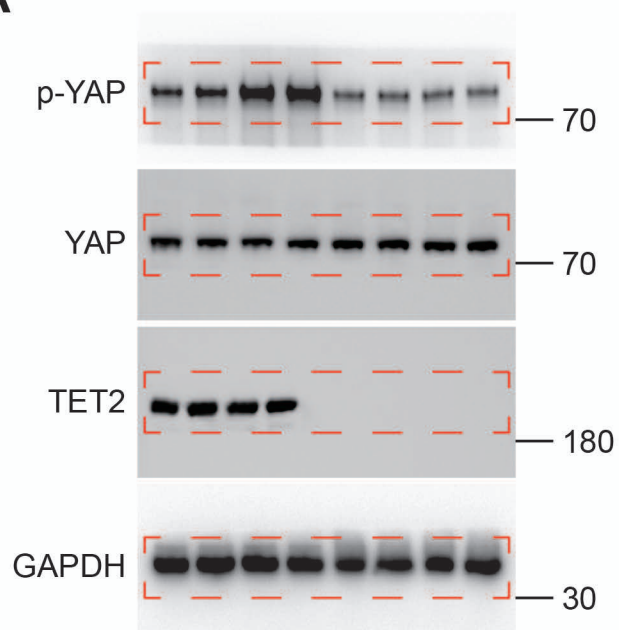

**E**

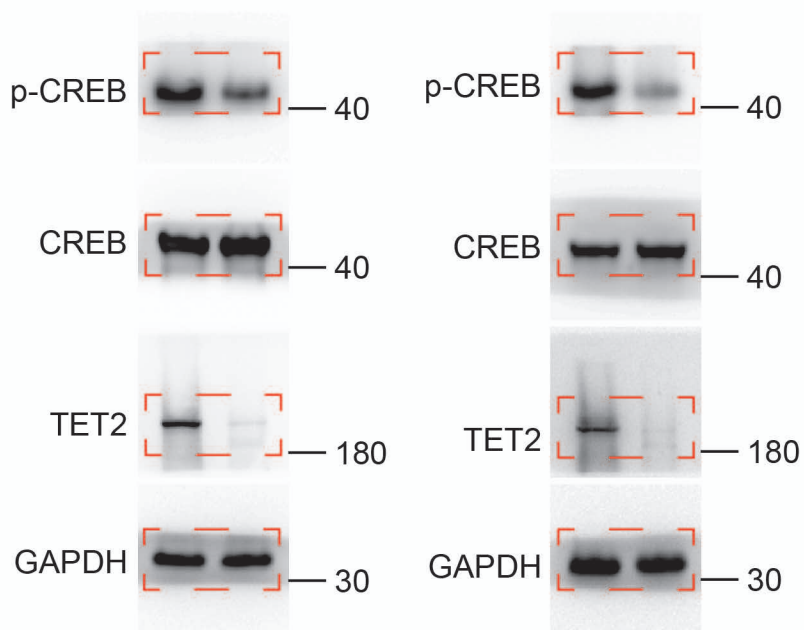

**F**

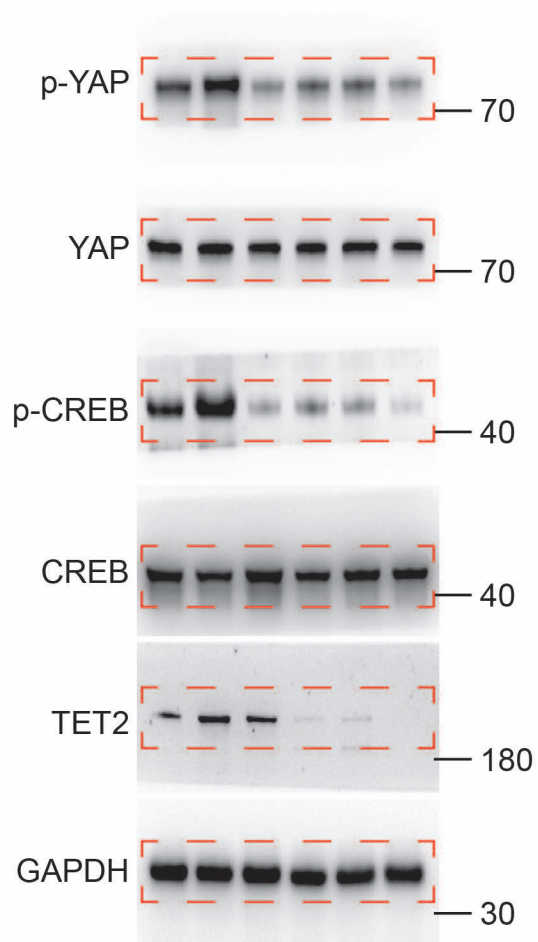

**J**

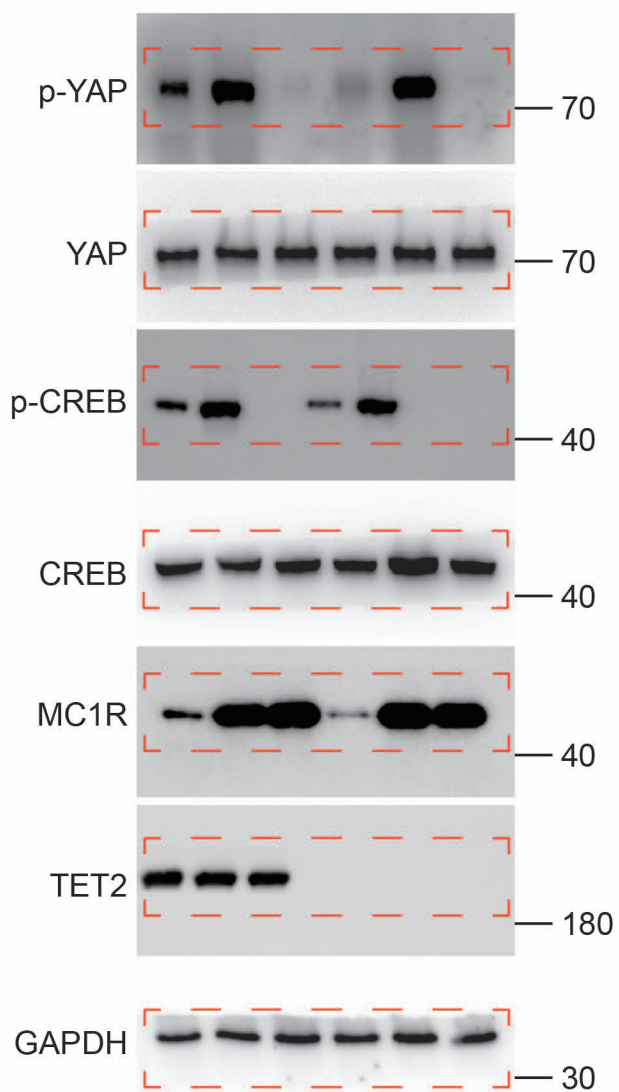

# Figure 3

**C**

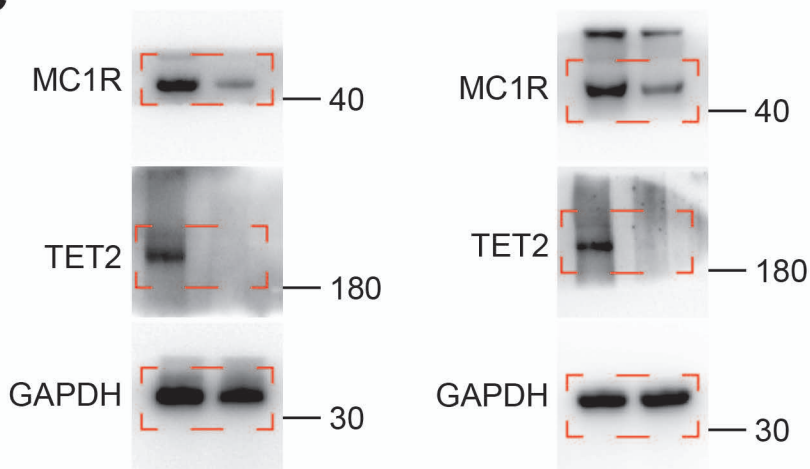

**D**

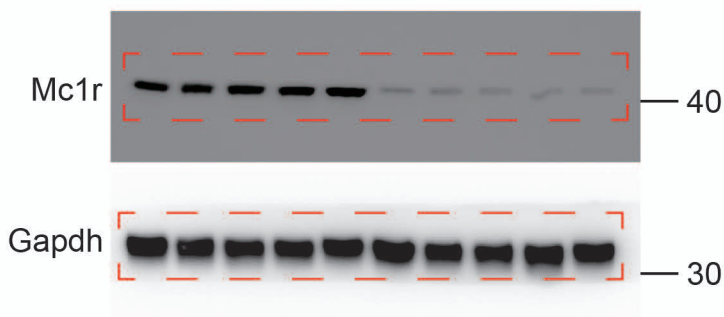

**G**

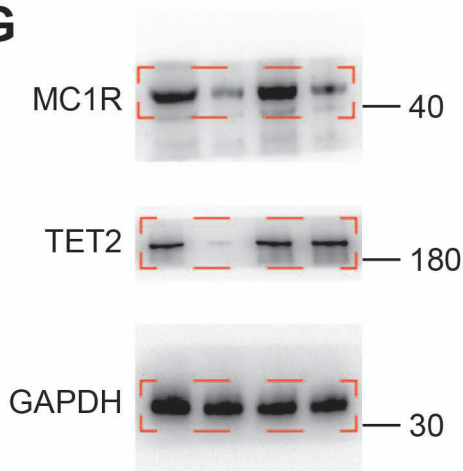

Figure 4

B

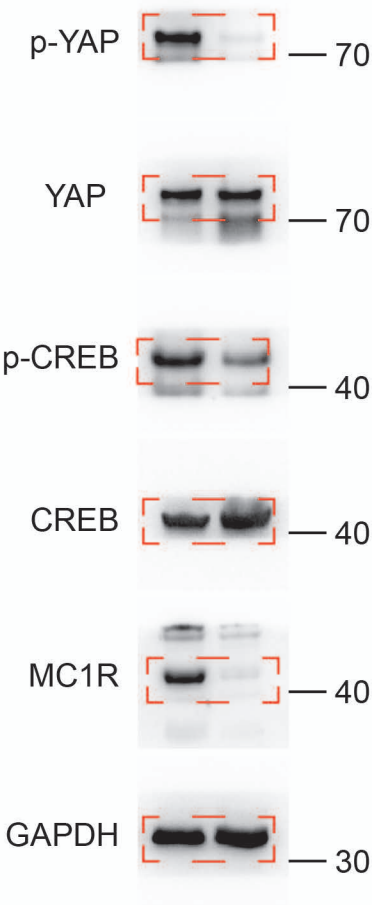

E

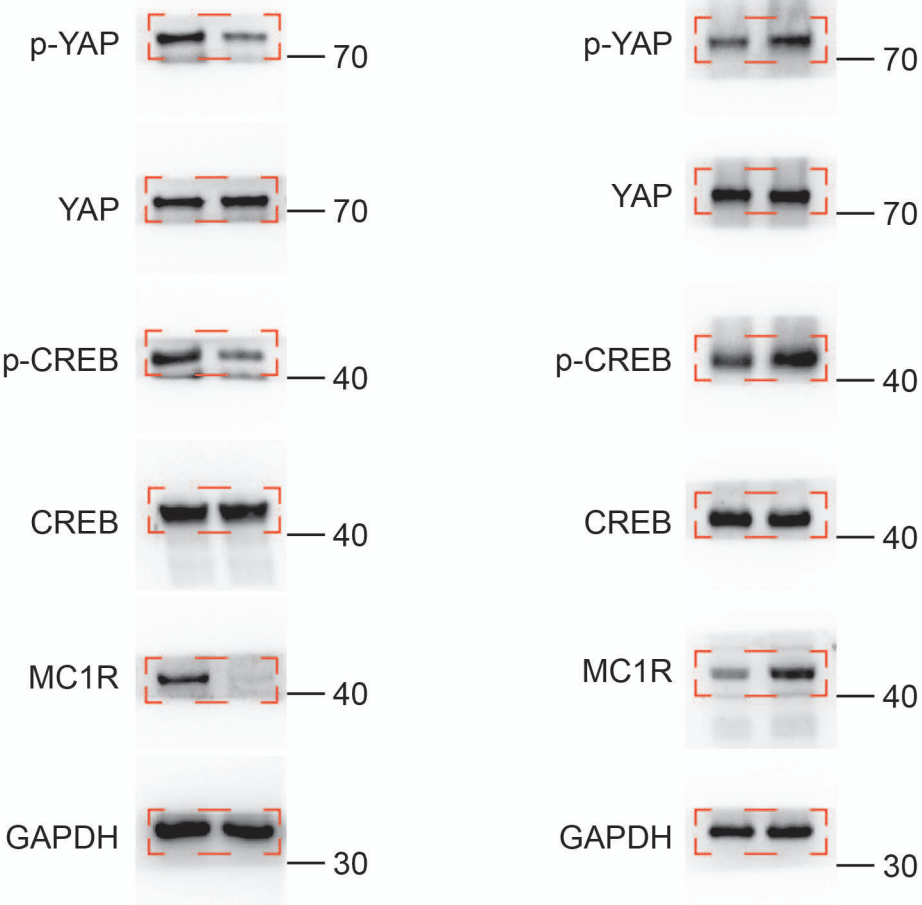

G

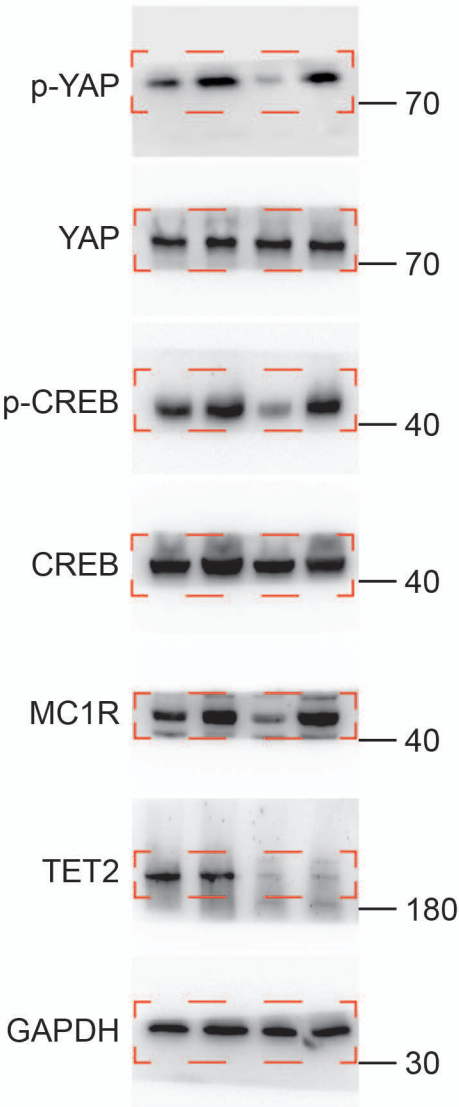

I

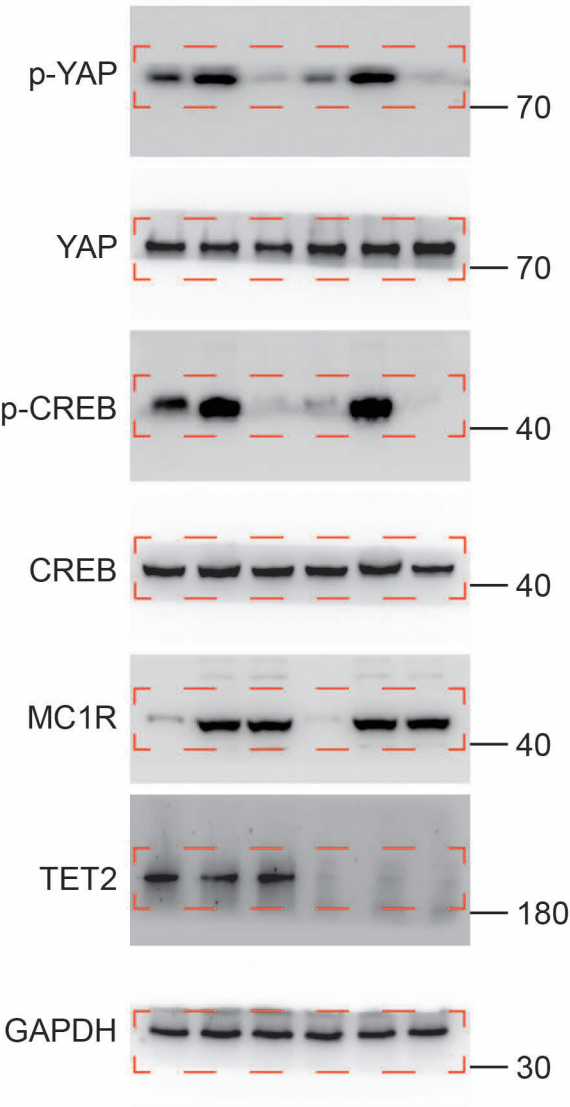

**Figure 6**

**A**

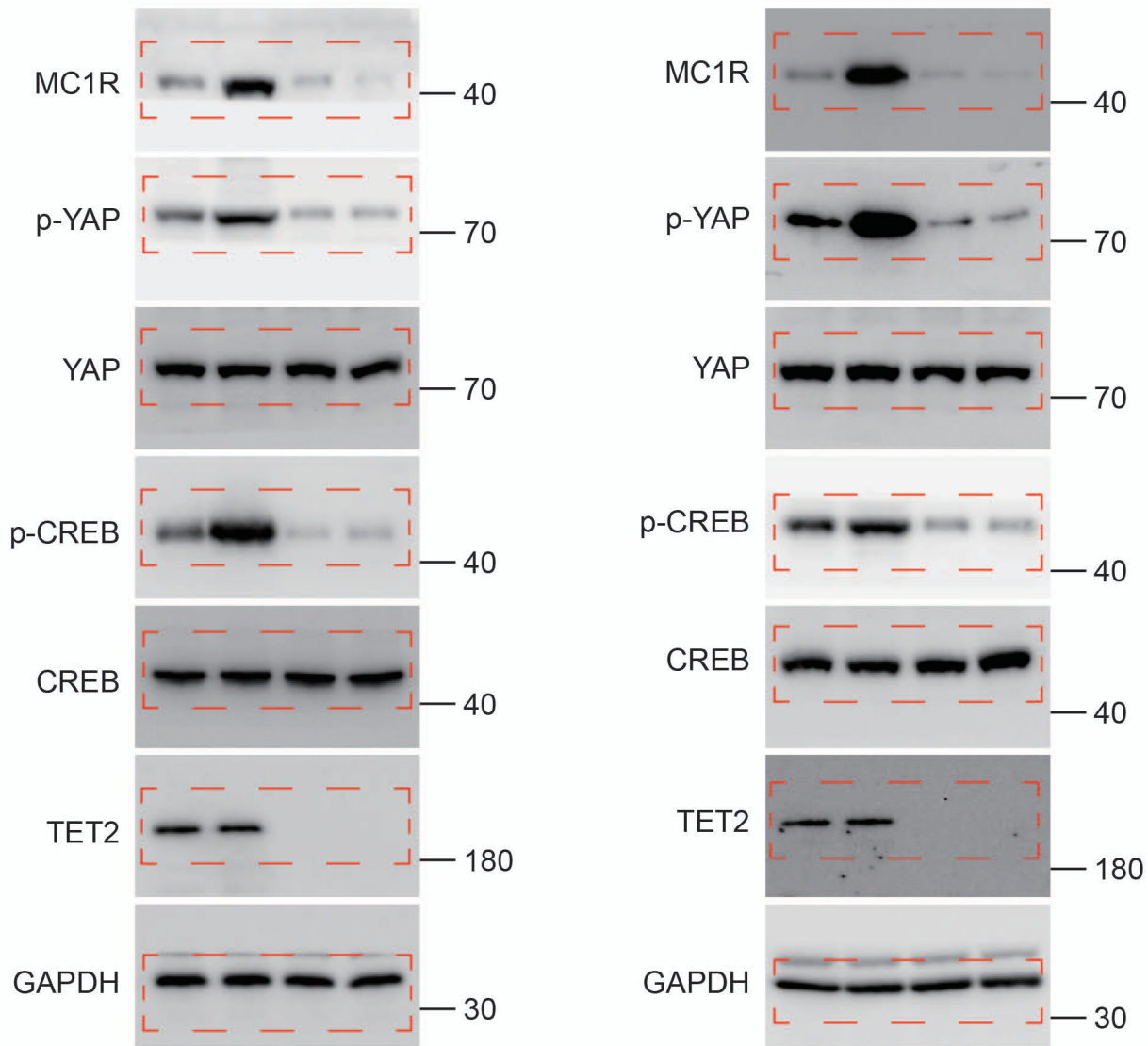

**B**

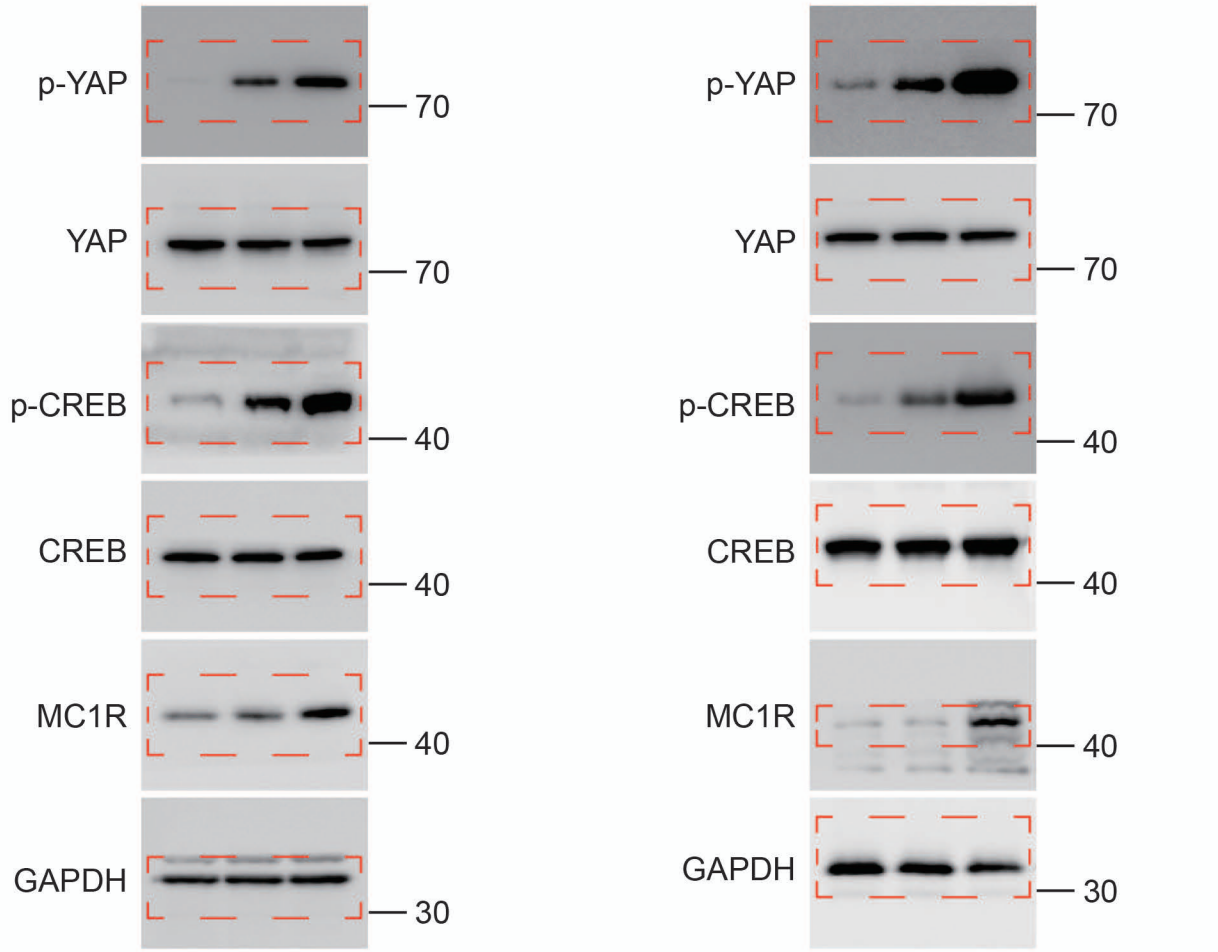

**Figure S1**

**B**

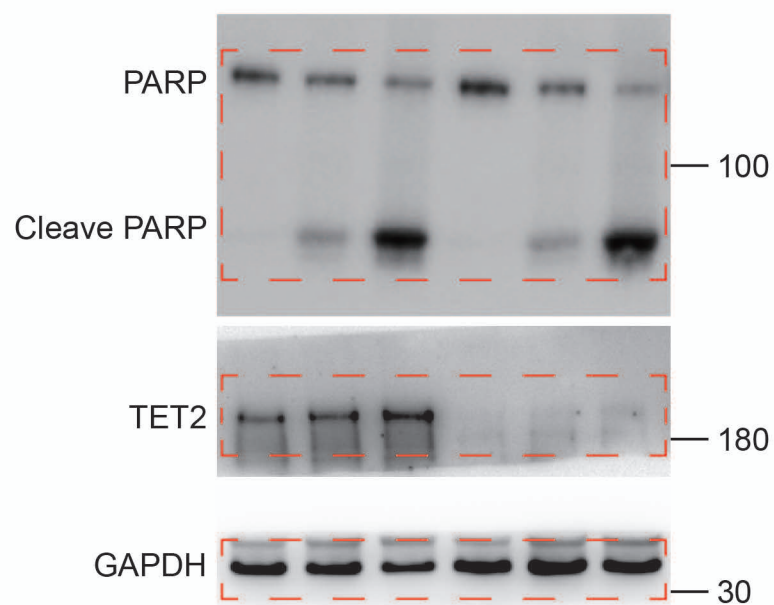

**C**

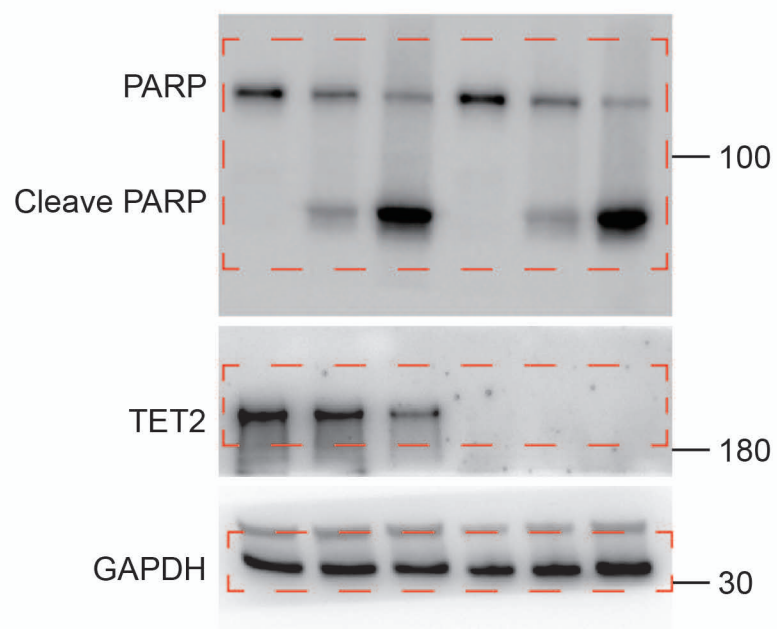

**D**

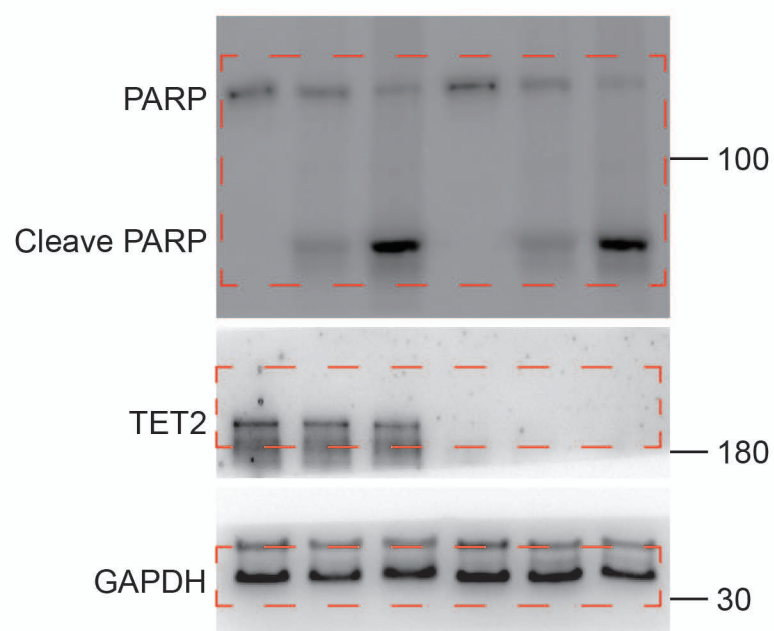

**E**

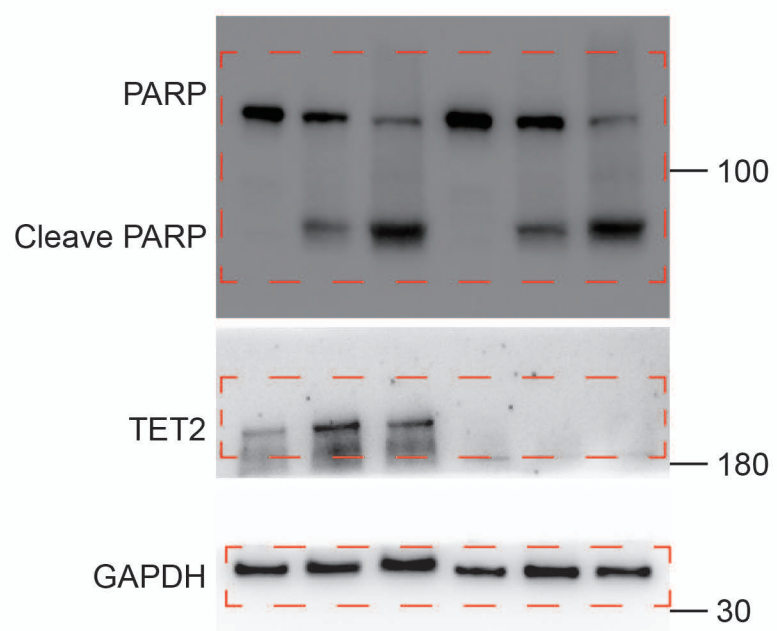

**F**

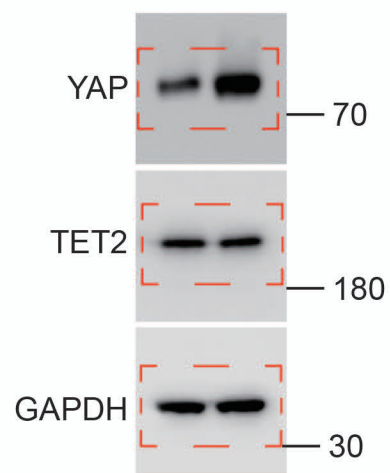

**G**

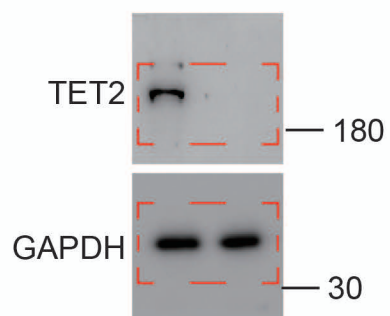

**Figure S2**

**A**

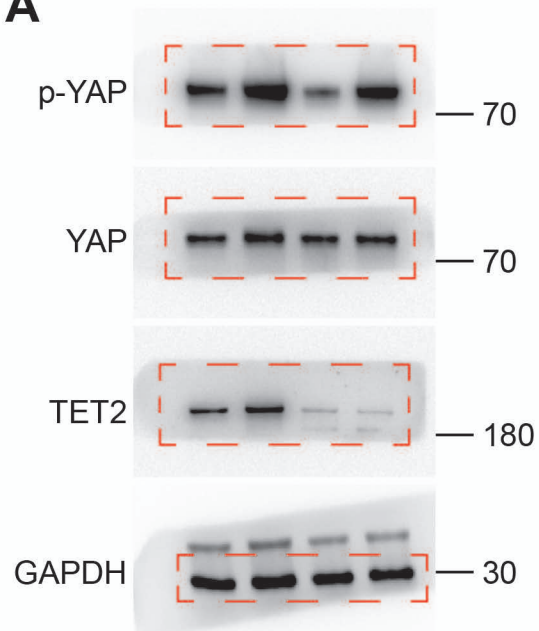

**B**

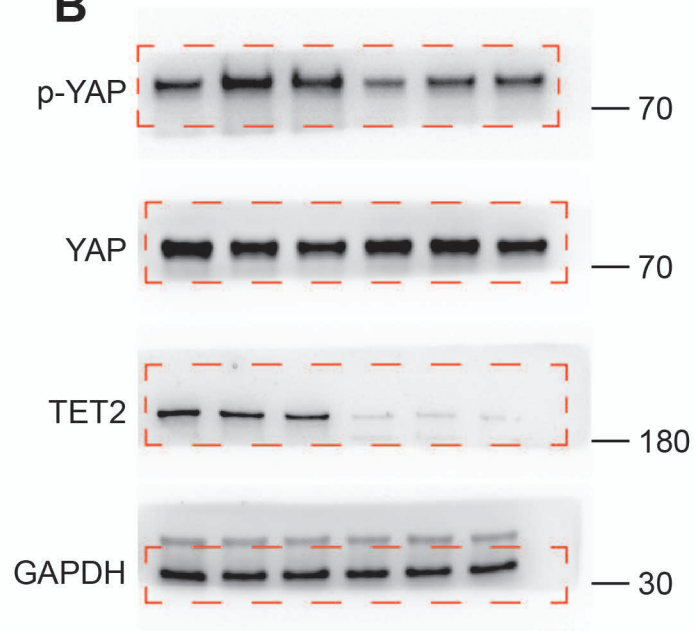

**C**

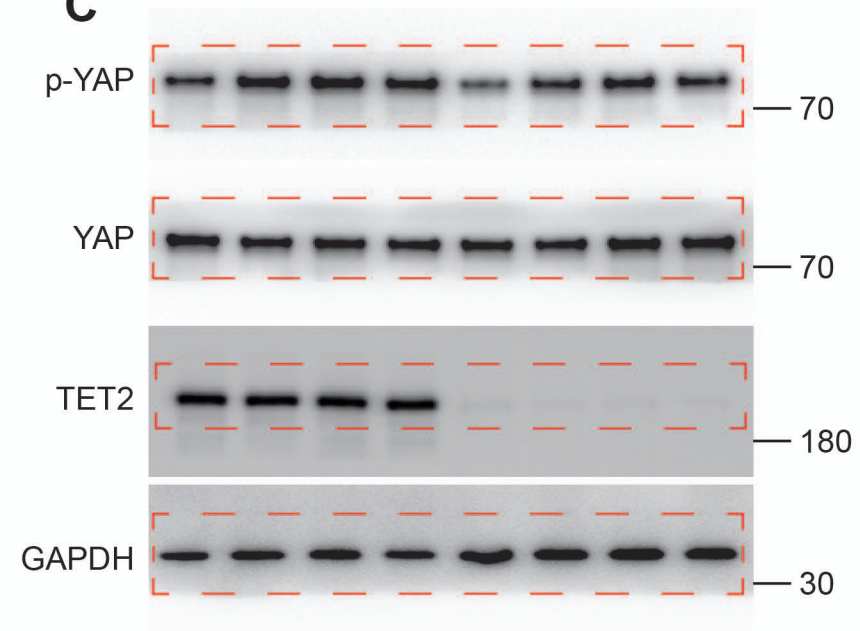

**D**

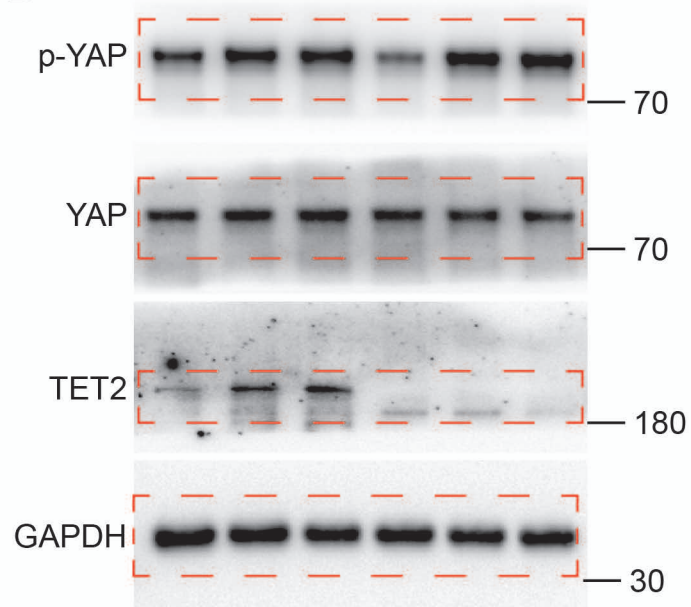

**E**

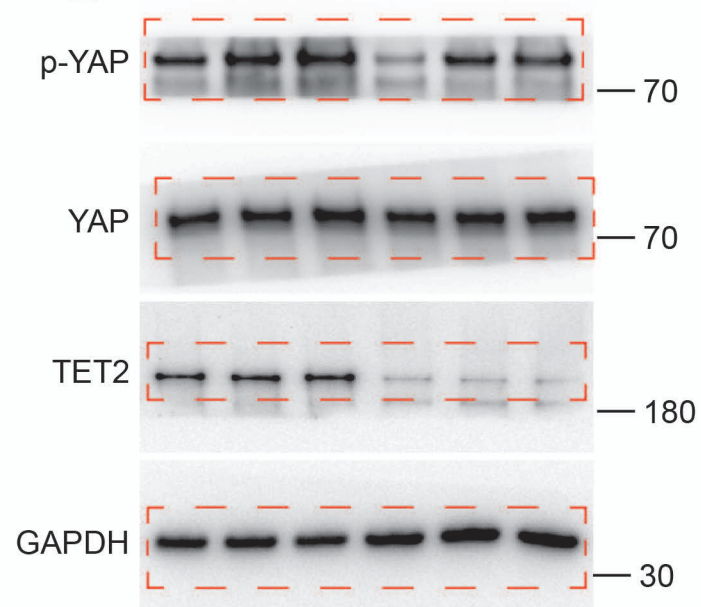

Supplement: Supplementary file 2 — Uncropped blots [file 41419_2025_7745_MOESM2_ESM.pdf]
